# *Lotus japonicus Nuclear Factor YA1*, a nodule emergence stage‐specific regulator of auxin signalling

**DOI:** 10.1111/nph.16950

**Published:** 2020-11-02

**Authors:** Arina Shrestha, Sihui Zhong, Jasmine Therrien, Terry Huebert, Shusei Sato, Terry Mun, Stig U. Andersen, Jens Stougaard, Agnes Lepage, Andreas Niebel, Loretta Ross, Krzysztof Szczyglowski

**Affiliations:** ^1^ Agriculture and Agri‐Food Canada London Research and Development Centre London ON N5V 4T3 Canada; ^2^ Department of Biology University of Western Ontario London ON N6A 5BF Canada; ^3^ Graduate School of Life Sciences Tohoku University 2‐1‐1 Katahira Sendai 980‐8577 Japan; ^4^ Department of Molecular Biology and Genetics Aarhus University Aarhus DK‐8000 Denmark; ^5^ Laboratoire des Interactions Plantes‐Microorganismes (LIPM) Université de Toulouse, Institut National de la Recherche pour l’Agriculture l’Alimentation et l’Environnement (INRAE) Centre National de la Recherche Scientifique (CNRS) Castanet‐Tolosan 31326 France

**Keywords:** auxin, legume, *Lotus*, *Medicago*, *NF‐YA1*, root nodule, symbiosis

## Abstract

- Organogenesis of legume root nodules begins with the nodulation factor‐dependent stimulation of compatible root cells to initiate divisions, signifying an early nodule primordium formation event. This is followed by cellular differentiation, including cell expansion and vascular bundle formation, and we previously showed that *Lotus japonicus NF‐YA1* is essential for this process, presumably by regulating three members of the *SHORT INTERNODES/STYLISH* (*STY*) transcription factor gene family.
- In this study, we used combined genetics, genomics and cell biology approaches to characterize the role of *STY* genes during root nodule formation and to test a hypothesis that they mediate nodule development by stimulating auxin signalling.
- We show here that *L*.* japonicus STY*s are required for nodule emergence. This is attributed to the *NF‐YA1*‐dependent regulatory cascade, comprising *STY* genes and their downstream targets, *YUCCA1* and *YUCCA11*, involved in a local auxin biosynthesis at the post‐initial cell division stage. An analogous *NF‐YA1/STY* regulatory module seems to operate in *Medicago truncatula* in association with the indeterminate nodule patterning.
- Our data define *L*.* japonicus* and *M*.* truncatula NF‐YA1* genes as important nodule emergence stage‐specific regulators of auxin signalling while indicating that the inductive stage and subsequent formation of early nodule primordia are mediated through an independent mechanism(s).

## Introduction

Symbiotic nodules develop as lateral organs on legume and some nonleguminous plant roots (Sprent & James, 2007; Doyle, 2011; Werner *et al*., 2014), usually following induction by nodulation factors (NFs), morphogenic lipo‐chitooligosaccharides synthesized by symbiotic rhizobia (Lerouge *et al*., 1990). Their perception by a compatible LysM‐type receptor kinase (Limpens *et al*., 2003; Madsen *et al*., 2003; Radutoiu *et al*., 2003, 2007; Arrighi *et al*., 2006; Broghammer *et al*., 2012; Liang *et al*., 2014; Kelly *et al*., 2017) induces local reprograming of root cells towards symbiotic development (Geurts *et al*., 2016; Wong *et al*., 2019). Despite commonalities with lateral root formation in both ontogeny (Herrbach *et al*., 2014; Xiao *et al*., 2019) and molecular mechanism (Schiessl *et al*., 2019; Soyano *et al*., 2019), nitrogen‐fixing root nodules are anatomically and functionally distinct organs (Oldroyd *et al*., 2009; Madsen *et al*., 2010).

Downstream from NF perception, cytokinins are endogenous inducers of root nodule formation (Cooper & Long, 1994; Gonzalez‐Rizzo *et al*., 2006; Murray *et al*., 2007; Tirichine *et al*., 2007; Plet *et al*., 2011; Ariel *et al*., 2012; Boivin *et al*., 2016; Miri *et al*., 2016; Gamas *et al*., 2017; Reid *et al*., 2017; H. Liu *et al*., 2018), distinguishing nodulation from lateral root evelopment, where auxin priming is required (Du & Scheres, 2018). A local increase in sensitivity to cytokinin in the root cortex was proposed as essential for the initiation of nodule primordia (NP) formation (Held *et al*., 2014). Given more recent data showing that legumes differ from nonlegumes in this respect, the acquisition of unique responsiveness to cytokinin by the root cortex might have contributed to legume‐specific diversification, perhaps underpinning the evolution of nodulation (Gauthier‐Coles *et al*., 2019).

Absence of nodulation in several plant lineages in the N_2_‐fixing clade, including legumes and nonlegumes, coincided with independent losses or pseudogenization of a limited number of symbiotic loci, including *NODULE INCEPTION* (*NIN*) (Griesmann *et al*., 2018; Van Velzen *et al*., 2018), which responds to cytokinin signalling in the root cortex (Vernie *et al*., 2015; Gamas *et al*., 2017; Murray, 2017). *NIN*, which encodes an RWP‐RK domain‐containing transcription regulator belonging to the NIN‐like protein (NLP) family (Chardin *et al*., 2014; Nishida *et al*., 2018), plays a complex role during N_2_‐fixing symbiosis. Essential in the epidermis for rhizobial entry via a root hair infection thread (IT)‐dependent mechanism (Schauser *et al*., 1999; Marsh *et al*., 2007; Kosuta *et al*., 2011; C. W. Liu, *et al*., 2019; Soyano *et al*., 2019), *NIN* also mediates the initiation of NP formation in the subtending root cortex and pericycle. Responsiveness of *NIN* to cytokinin is critical in the latter context (Heckmann *et al*., 2011; Yoro *et al*., 2014; J. Liu *et al*., 2019) and its overexpression in both *L*.* japonicus* and *M*.* truncatula* roots was sufficient for pseudonodule formation, mimicking the effect of NFs and cytokinins (Soyano *et al*., 2013; Vernie *et al*., 2015).

Several direct NIN targets, including *LOB‐Domain Protein 16*/*Asymmetric Leaves 2‐Like 18* (*LBD16*/*ASL18*), *Nuclear Factor‐YA1* (*NF‐YA1*) and *NF‐YB1*, have been identified as relevant to NP formation (Soyano *et al*., 2013, 2019; Schiessl *et al*., 2019). *NIN*‐dependent activation of the cytokinin responsive *LBD16*/*ASL18* promoted cell divisions during early NP formation (Schiessl *et al*., 2019) and *L*.* japonicus* LBD16/ASL18 was shown to interact with NF‐YA1 *in vitro* and also *in vivo* when ectopically expressed *in planta* (Soyano *et al*., 2019).

NF‐Ys are heterotrimeric transcription factors comprising NF‐YA, NF‐YB, and NF‐YC subunits (Mantovani 1999; Laloum *et al*., 2013) that are essential during rhizobial infection and nodule formation (Combier *et al*., 2006, 2008; Zanetti *et al*., 2010; Soyano *et al*., 2013; Battaglia *et al*., 2014; Laloum *et al*., 2014; Laporte *et al*., 2014; Baudin *et al*., 2015; Hossain *et al*., 2016; Zanetti *et al*., 2017; Ripodas *et al*., 2019; Bu *et al.*, 2020). Ectopic expression of *NF‐YA1* and *NF‐YB1*, along with *LBD16/ASL18*, coordinately stimulated cortical cell divisions and partially rescued the defective nodulation phenotype of *L*.* japonicus daphne*, which carries a mutant *NIN* allele, suggesting that their interaction is important during nodule initiation (Soyano *et al*., 2013, 2019; Yoro *et al*., 2014). However, we showed that *L*.* japonicus NF‐YA1* was dispensable at this early developmental phase yet essential for nodule differentiation, including cell expansion and vascular bundle formation, presumably through regulation of three *SHORT INTERNODES/STYLISH* (*STY*) transcription factor genes, *STY1*, *STY2* and *STY3* (Hossain *et al*., 2016). STYs are known to regulate auxin homeostasis (Sohlberg *et al*., 2006; Eklund *et al*., 2010a,b; Baylis *et al*., 2013; Estornell *et al*., 2018) and we postulated a similar function during nodule differentiation (Hossain *et al*., 2016).

We demonstrate here that upon inoculation with *Mesorhizobium loti*, *L*.* japonicus NF‐YA1* facilitates the expression of seven *STY*s that regulate at least two *YUCCA* genes involved in auxin biosynthesis. The *STY* genes, like *NF‐YA1*, proved to be essential for nodule emergence but were also crucial for *M*.* loti* infection. A partial, functional redundancy was found between *NF‐YA1* and *NF‐YA4*. The *nf‐ya1 nf‐ya4* double mutant, unable to regulate the *STY* and *YUCCA* gene expression in response to *M*.* loti* infection, developed many small primordia that did not differentiate into nodules, a phenotype that persisted in *nf‐ya* triple and quadruple mutant lines. These results indicate that initiation of cell divisions for NP does not require *NF‐YA1* and other partially redundantly acting *NF‐YA*s. However, *NF‐YA1* and *STY*s are indispensable during the nodule emergence stage. An equivalent *M*.* truncatula NF‐YA1*/*STY* regulatory circuit probably partakes in mediation of the patterning of indeterminate nodules.

## Materials and Methods

### Plant materials, growth conditions and assessment of phenotypes

Mutants were identified using the *L*.* japonicus* LORE1 retrotransposon insertion line collection at Aarhus University in Denmark (https://lotus.au.dk/; Mun *et al*., 2016). Plants were genotyped using the gene‐ and LORE1‐specific primers, following established procedures (Urbanski *et al*., 2012).

For the *STY3* locus, additional mutant alleles, called *sty3‐1* to *sty3‐9*, were identified using the *L*.* japonicus* Targeting Induced Local Lesions In Genomes (TILLING) resource at the John Innes Centre (RevGenUK; https://www.jic.ac.uk/technologies/genomic‐services/revgenuk‐tilling‐reverse‐genetics/; Perry *et al*., 2009). Two homozygous lines, *sty3‐1* and *sty3‐9*, with predicted premature termination codons were genotyped using derived cleavage amplified polymorphic sequence markers, followed by enzymatic digest with either *Age*I (*sty3‐1*) or *Pvu*II (*sty3‐9*) (Table S1). Higher‐order mutant lines were developed by performing genetic crosses between selected homozygous single and double mutants. The F_3_ progeny derived from homozygous higher‐order mutant lines were utilized for subsequent phenotypic analyses.

Germination, growth conditions and assessment of symbiotic phenotypes were as previously described (Wopereis *et al*., 2000). For nonsymbiotic phenotypic analyses, vermiculite : sand (6 : 1) growth medium was supplemented with Broughton & Dilworth solution containing 1 mM KNO_3_.

For gene expression analysis (quantitative reverse transcription polymerase chain reaction (qRT‐PCR)), *L*. *japonicus* seedlings were grown in sterile conditions for 7 d and inoculated with *M*.* loti*. Control uninoculated roots were harvested at this time, while inoculated roots were harvested 4, 7, 12 or 21 d later. At least three independent biological replicates per treatment were collected. For gene expression analysis in the primary transformants, T0 plants, *STY3::SRDX5* and *STY3::SRDX6* (see below) were propagated by cuttings, as follows: shoot tip cuttings of *c*. 4 cm in length from branches of T0 plants, along with control wild‐type cuttings, were set into a vermiculite : sand (6 : 1) mixture soaked in B&D solution containing 0.5 mM KNO_3_, where they were grown for 14 d under sterile conditions and then inoculated with *M*.* loti*.

### STY3::SRDX dominant negative constructs

The *NF‐YA1_Pro_:STY3::SRDX:NF‐YA1‐3*′*UTR* binary vector (Hossain *et al*., 2016) was transferred in parallel with an empty pKGWD,0 vector, used as a negative control, to *Agrobacterium tumefaciens* strain LBA4404. Standard transformation protocols were employed to generate fully transgenic *L*.* japonicus* plants (Lombari *et al*., 2005) The primary transgenic T0 plants were allowed to self‐fertilize and the resulting T1 populations were genotyped for the *NF‐YA1_Pro_:STY3::SRDX* transgene (Table S1) and evaluated for nodulation phenotypes.

### Gene expression analysis using qRT‐PCR

Total RNA was extracted from roots using the Plant/Fungi Total RNA Purification Kit (Norgen Biotek Corp., Thorold, ON, Canada), treated with TURBO DNase I (Invitrogen) and assessed for purity and quality. cDNA was prepared from 500 ng of RNA using the SuperScript IV VILO Master Mix (Invitrogen). qRT‐PCR was performed using three to five biological and three technical replicates, on a CFX384 Real‐Time PCR Detection System (Bio‐Rad, Mississauga, ON, Canada) using the SensiFAST SYBR No‐ROX kit (Bioline, Memphis, TN, USA) under the following conditions: 95°C for 3 min followed by 40 cycles of 95°C for 5 s, 60°C for 15 s and 72°C for 15s. Expression levels were normalized against three reference genes (UBQ, PP2A and ATP‐s) as previously described (Held *et al*., 2014). Primer sequences used for the qRT‐PCR expression analyses are listed in Table S1.

### Gene promoter activity localization using GUS histochemical assay

To develop the localization constructs, fragments encompassing the *STY1* to *STY9* promoter regions, were prepared by gene synthesis (Bio Basic Inc., Markham, ON, Canada). The promoter sequences were synthesized as such, for *STY1* (position −3422 to −1), *STY2* (−4000 to −1), *STY3* (−4000 to −1), *STY4* (−4000 to −1), *STY5* (−3000 to −1), *STY6* (−2177 to −1), *STY7* (−4000 to −1), *STY8* (−4000 to −1), and *STY9* (−2487 to −1), where −1 denotes the first base upstream from predicted ATG initiation codons. These were recombined in the pKGWFS7 destination vector containing an in‐frame fusion between the regions coding for green fluorescent protein (GFP)/β‐glucuronidase (GUS) (Karimi *et al*., 2002; https://gateway.psb.ugent.be/), using the Gateway technology (Invitrogen).

The TAC/BAC clones, LjT09B24 (TM2451) and LjB04P03 (BM2658) (http://www.kazusa.or.jp/lotus/), were used to amplify the promoter regions of *YUCCA1* (position −2466 to −1) and *YUCCA11* (−2249 to −1), respectively. The resulting promoter fragments were cloned into the pENTR/D‐TOPO vector (Invitrogen) and subsequently recombined in the pKGWFS7 destination vector.

After validation by sequencing, constructs were transformed into *Agrobacterium rhizogenes* strains AR1193 and ARquA1 for *Lotus* and *Medicago* hairy root transformation, respectively. At least 10 independent hairy root systems were analysed for GUS reporter activity, following an established protocol (Held *et al*., 2014). Histochemical assays of *M*.* truncatula* hairy roots were performed as previously described (Cerri *et al*., 2012). Magenta‐Gal (5‐bromo‐6‐chloro‐3‐indolyl‐β‐d‐galactopyranoside; Biosynth B7200) and X‐Gluc (5‐bromo‐4‐chloro‐3‐indolyl‐β‐d‐glucuronic acid, cyclohexylammonium salt; Thermo Scientific, Waltham, MA, USA) substrates were used for histochemical staining of *Sinorhizobium meliloti* (β‐galactosidase; purple color) and GUS (blue color) activity, respectively, within ITs and nodules.

### YUCCA1 and YUCCA11 overexpression experiments

The *YUCCA1* and *YUCCA11* overexpression constructs were prepared by synthesizing a 769 bp long DNA fragment encompassing the sequence of the 2 × *Cauliflower mosaic virus* 35S promoter with attL1 and attR5 recombination sites at its 5′ and 3′ ends, respectively (Bio Basic Inc.). The *YUCCA1* cDNA and *YUCCA11* genomic sequences were likewise synthesized. The former encompassed the entire 1251bplong coding region, predicted 5′‐ (201bp) and 3′‐ (585bp) UTRs, and attL5 and attL2 at 5′ and 3′ ends, respectively. The *YUCCA11* genomic region included all exons and introns (1896 bp long, from ATG to the predicted stop codon), 500 bp 3′UTR and attL5 and attL2 recombination sites.

These DNA fragments were recombined into the pKGWD,0 gateway destination vector to generate *2X35S_Pro_:YUCCA1* and *2X35S_Pro_:YUCCA11*. They were transformed into *A*.* rhizogenes* strain AR1193, which was used to generate hairy roots on transgenic, *L*.* japonicus* shoots carrying the *DR5*:*GUS* reporter.

### Development of *DR5:GUS* reporter line in *nf‐ya1‐2 nf‐ya4* background

The transgenic *L*.* japonicus* line carrying the *DR5:GUS* reporter was crossed with homozygous *nf‐ya1‐2 nf‐ya4*. The F_1_ plants were allowed to self‐fertilize to produce segregating F_2_ populations, and homozygous *nf‐ya1‐2 nf‐ya4* plants carrying *DR5:GUS* were selected for propagation.

### Next‐generation RNA sequencing

*Mesorhizobium loti*‐inoculated root samples were harvested 4 d after inoculation (dai), along with corresponding uninoculated roots of the same age. Total RNA was extracted using the Plant/Fungi Total RNA Purification Kit (Norgen Biotek Corp., Thorold, ON, Canada). RNA quality was verified using an Agilent Bioanalyzer 2100 (Agilent Technologies, Santa Clara, CA, USA). The RNA library was constructed and sequenced at the Center for Applied Genomics at Sick Kids Hospital (Toronto, Canada) using Illumina Hi‐Seq 2500 paired‐end reads. Mapping of reads to the *L*.* japonicus* MG20 v.3.0 genome was performed using clc genomics workbench with the following parameters: mapping type: map to gene regions only; mismatch cost: 2; insertion cost: 3; deletion cost: 3; length fraction: 0.8; similarity fraction: 0.8; global alignment: no; auto‐detect paired distances: yes; strand‐specific: both; maximum number of hits for a read: 10; count paired reads as two: no; expression value: reads per kilobase of transcript, per million mapped reads (RPKM) and transcripts per kilobase million (TPM); calculate RPKM and TPM for genes without transcripts: no; use EM estimation: yes; create fusion gene table: no. After the expression analysis, Gaussian statistical analysis was performed on all the tables using the *t*‐test function, and the *P*‐values were false discovery rate (FDR)‐corrected.

### Phylogenetic analyses and microscopy

Protein sequences were aligned with clustalw and the corresponding phylogenetic trees were generated using Mega 7 software and the neighbour‐joining method with bootstrap replicates of 1000. All microscopic observations were performed using either a Nikon Eclipse Ni upright or Nikon SMZ25 stereo microscope integrated with a DsRi2 digital camera and epifluorescence capability (Nikon, Tokyo, Japan). All images were captured in a TIFF format and subsequently processed using Adobe photoshop cs6.

## Results

### Activities of *L*.* japonicus* and *M*.* truncatula SHI/STY* genes associate with root and nodule development

**Fig. 1:**
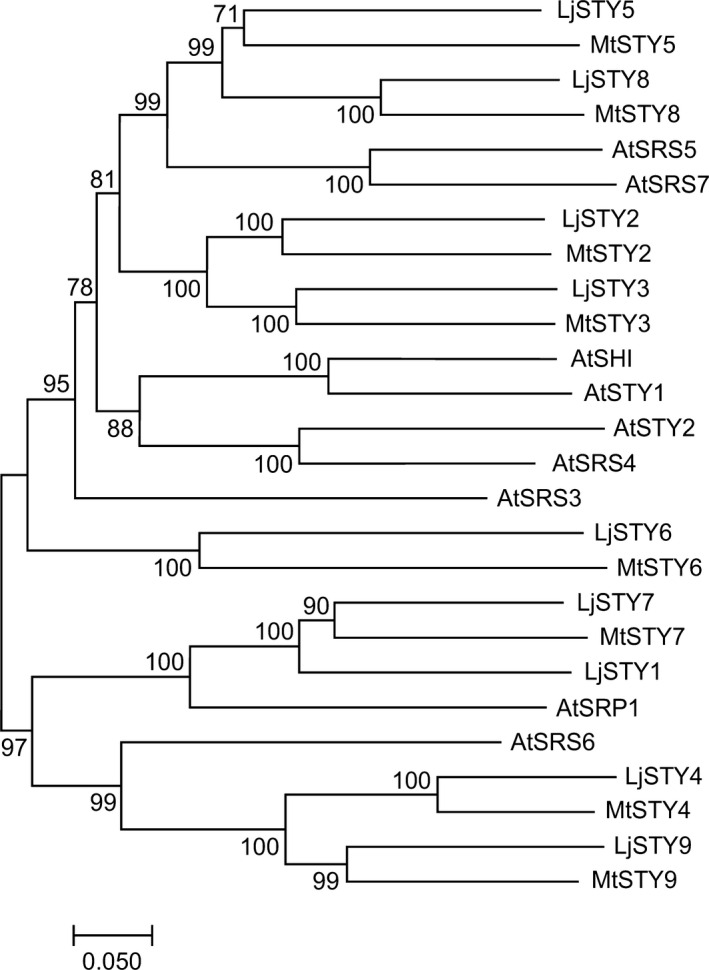
Phylogenetic relationship between predicted STY proteins from *Lotus japonicus*, *Medicago truncatula* and *Arabidopsis thaliana*. The unrooted tree was constructed using full‐length protein sequences. For the sake of coherence the same number was given to *Medicago* and *Lotus STY* genes when a clear homologue was present in both species. The following accession numbers refer to the protein sequences used: *L*.* japonicus* – LjSTY1 (Lj6g3v0959410), LjSTY2 (Lj0g3v0059359), LjSTY3 (Lj2g3v1728900), LjSTY4 (Lj3g3v0766120), LjSTY5 (Lj1g3v2140900), LjSTY6 (Lj3g3v3376040), LjSTY7 (Lj2g3v3044220), LjSTY8 (Lj5g3v0155490)¸ and LjSTY9 (Lj0g3v0258549); *M*.* truncatula* – MtSTY2 (MtrunA17Chr8g0372461), MtSTY3 (MtrunA17Chr5g0404781), MtSTY4 (MtrunA17Chr3g0082511), MtSTY5 (MtrunA17Chr3g0142171), MtSTY6 (MtrunA17Chr4g0035591), MTSTY7 (MtrunA17Chr5g0441921), MtSTY8 (MtrunA17Chr1g0155791) MtSTY9 (MtrunA17Chr8g0353111); and *A*.* thaliana* SHI (At5g66350), STY1 (NM_114966), STY2 (At4g36260), LRP1 (At5g12330), SRS3 (At2g21400), SRS4 (At2g18120), SRS5 (At1g75520), SRS6 (At3g54430), SRS7 (At1g19790), SRS8 (At5g33210).

Using *L*.* japonicus* STY1, STY2 and STY3 and *A*.* thaliana* SHI/STY protein sequences as queries, nonredundant sequence collections at the NCBI server (https://www.ncbi.nlm.nih.gov/) and the *L*.* japonicus* (www.kazusa.or.jp/lotus/; https://lotus.au.dk/) and *M*.* truncatula* (https://medicago.toulouse.inra.fr/MtrunA17r5.0‐ANR/) databases were analysed. Predicted *L*.* japonicus* and *M*.* truncatula SHI/STY* gene families of nine and eight members, respectively, were identified (Fig. 1). All encoded proteins contained the evolutionarily conserved RING‐type zinc finger (IPR001841) and IGGH domains, the latter probably specific to the SHI/STY protein family (Fridborg *et al*., 2001; Eklund *et al*., 2010b; Gomariz‐Fernandez *et al*., 2017) (Figs S1–S3).

Follow‐up experiments were directed toward functional characterization of the *L*.* japonicus* genes but limited comparative studies with *M*.* truncatula STY*s were also performed. *Lotus japonicus* and *M*.* truncatula* form determinate and indeterminate (i.e. meristem containing) nodules, respectively (Szczyglowski *et al*., 1998; Sprent & James, 2007; Xiao *et al*., 2014), which both require *NF‐YA1* (Combier *et al*., 2006, 2008; Soyano *et al*., 2013), and we showed previously that *MtNF‐YA1* can functionally replace *LjNF‐YA1* during nodule formation (Hossain *et al*., 2016). We have postulated, therefore, that an equivalent *NF‐YA1*/*STY* regulatory module must operate and be relevant during the indeterminate nodule development.

**Fig. 2:**
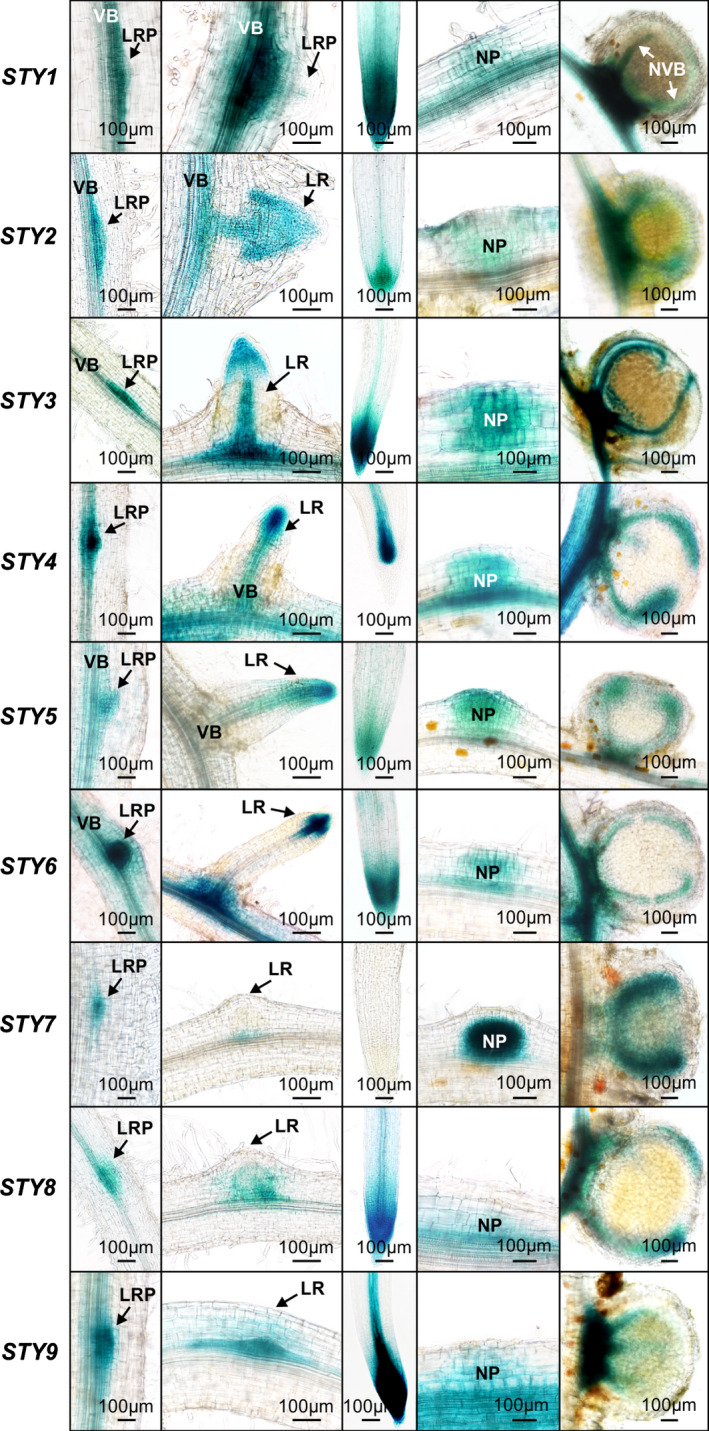
The activity of *Lotus japonicus STY* promoters during root and nodule development. Representative images of hairy root segments are shown. Columns from left to right depict the β‐glucuronidase (GUS) activity (in blue) as associated with small lateral root primordia (LRP), emerging or already emerged lateral roots (LR), root apical regions, nodule primordia (NP) and fully mature nodules. All specimens were collected 10–14 d after inoculation with *Mesorhizobium loti*. For each promoter:GUS construct, 10–15 individual hairy roots were analysed. VB, root vascular bundle; NVB, nodule vascular bundle.

Promoter‐GUS reporter constructs were developed for each *LjSTY* gene and their activities were tested in *A*.* rhizogenes*‐induced hairy roots (Fig. 2). Similar to *Arabidopsis* (Kuusk *et al*., 2002, 2006; Eklund *et al*., 2011), the GUS reporter activity was associated with early lateral root primordia, including the initial pericycle cell divisions and the central vasculature and root apices of emerged lateral roots. These activity patterns were shared by all *LjSTY* promoters, except for *LjSTY7*, for which GUS staining was only weakly detectable at the base of emerging lateral roots. Remarkably, all *STY* promoters were active in dividing cortical cells of young NP and later, at the base and in the vasculature of mature nodules (Fig. 2), resembling expression domains of *NF‐YA1* (Hossain *et al*., 2016).

**Fig. 3:**
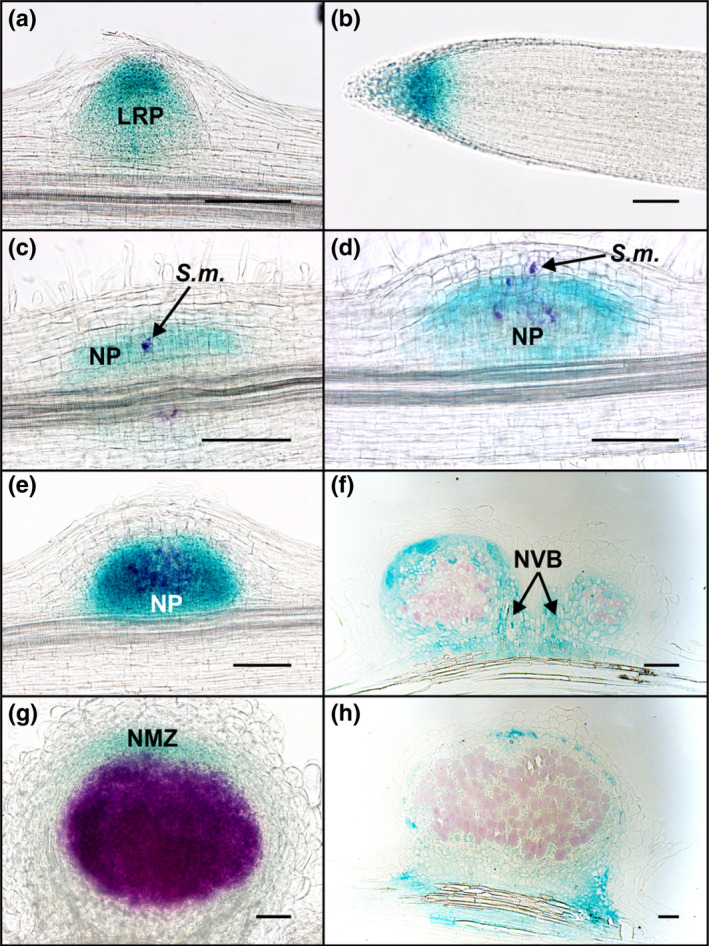
Expression analysis of *MtSTY2* during the symbiotic interaction between *Medicago truncatula* and *Sinorhizobium meliloti*. Roots expressing a *pMtSTY2:GUS* fusion were analysed at different stages after inoculation with *S*.* meliloti*. Double staining using Magenta‐Gal and X‐Gluc was performed to allow the visualization of the infecting *S*.* meliloti* in purple and *pMtSTY2* expression in blue. (a) *pMtSTY2:GUS* expression in a lateral root primordium (LRP) just before its emergence. (b) Expression in the meristematic zone of a main root. (c) *pMtSTY2:GUS* expression in dividing cells of the inner and central cortex of a 2‐d‐old nodule primordium. (d) *pMtSTY2:GUS* expression in dividing cells of a 3‐d‐old nodule primordium (NP). (e) *pMtSTY2:GUS* expression in the central zone of a 5‐d‐old nodule. (f) Thin section through a 5‐d‐old nodule. (g) *pMtSTY2:GUS* expression in a 10‐d‐old nodule. (h) Thin section through a 12‐d‐old nodule. Bars, 100 μm. *S.m.*, *Sinorhizobium meliloti*; NVB, nodule vascular bundle; NMZ, nodule meristematic zone.

We also explored the expression pattern of *M*.* truncatula STY2* (*MtSTY2*) using a 3089 bp promoter fragment fused to the *GUS* reporter gene. *MtSTY2* was chosen because its mRNA had one of the highest steady‐state levels in nodules (Roux et al., 2014). Similar to *L*.* japonicus STY*s, the *MtSTY2* promoter activity was localized primarily in the apical region of growing lateral root primordia and main roots (Fig. 3a,b). Upon *S*.* meliloti* infection, GUS activity was detected concurrent with early cell divisions associated with penetrating ITs in the inner root cortex (Fig. 3c,d). In young primordia and emerging nodules, GUS staining was apparent in most dividing cells (Fig. 3e), which was reminiscent of *L*.* japonicus* nodules (e.g. *LjSTY7*; Fig. 2). However, with further development, the reporter gene expression rapidly became restricted to uninfected cells of the nodule parenchyma and vascular cambium (Fig. 3f) and, later, to the nodule meristematic zone (Fig. 3g), often developing a patchy pattern as nodules grew older (Fig. 3h).

### The level of *STY* mRNAs is regulated during nodule development

**Fig. 4:**
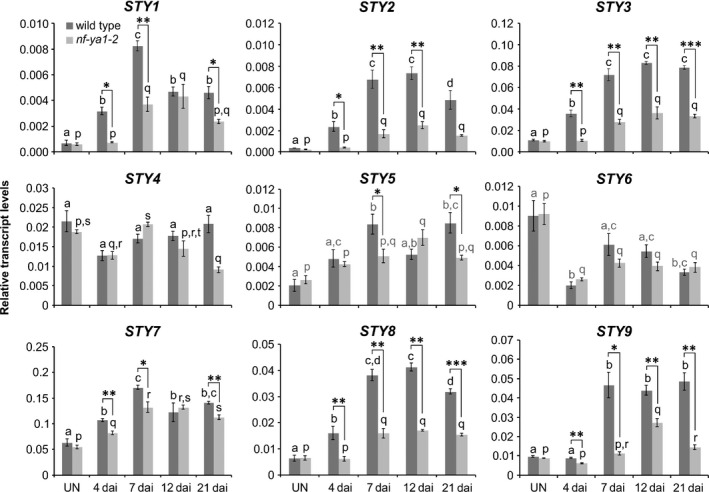
Expression of *Lotus japonicus STY* genes is regulated upon *Mesorhizobium loti* infection. Quantitative reverse transcription polymerase chain reaction data showing steady‐state levels of *Lotus* *japonicus STY* mRNAs in control, uninoculated (UN) roots of *L*.* japonicus* wild‐type (dark grey) and the *nf‐ya1‐2* mutant (light grey) and in corresponding root samples collected at various time points (d) after inoculation (dai) with *M*.* loti*. The means ± SE are given for three biological replicates. Statistical groupings across different time points, reflected by the same letters, have been determined separately for each genotype using one‐way ANOVA with the Tukey’s honestly significant difference *post hoc* test (*P* < 0.05). Student’s *t*‐test was used to carry out pairwise comparisons between the wild‐type and *nf‐ya1‐2* at each time point (*, *P* < 0.05; **, *P* < 0.01; ***, *P* < 0.001).

*STY* gene expression was further characterized through *in silico* analysis of our next‐generation RNA sequencing (RNA‐seq) data (BioProject ID PRJNA630938; http://www.ncbi.nlm.nih.gov/bioproject/630938). At 4 dai with *M*.* loti*, the steady‐state levels of *LjSTY1*, *2*, *3* and *7* mRNAs were significantly upregulated (Table S2). As the histochemical data indicated activity of all *LjSTY* promoters during nodule formation, the steady‐state level of the nine *LjSTY* mRNAs was further evaluated across four developmental stages (Fig. 4). Equivalent RNA samples derived from roots of *nf‐ya1‐2* (Hossain *et al*., 2016) were analysed in parallel in order to determine whether *STY* genes are regulated by *NF‐YA1* upon *M*.* loti* infection.

Consistent with the RNA sequencing results, at 4 dai, *STY1*, *2*, *3*, *7* and additionally *STY8* mRNAs were significantly upregulated in wild‐type roots, above control levels. This upregulation was lost in *nf‐ya1‐2*, except for *STY7*, which showed only a partial dependency on *NF‐YA1* (Fig. 4).

At 7 dai, levels of these five *STY* mRNAs were further enhanced and two additional *STY* mRNAs, *STY5* and *STY9*, were also significantly upregulated (Fig. 4). Interestingly, the initially upregulated *STY* mRNAs (i.e. *STY1*, *2*, *3*, *7* and *8*) displayed only a partial dependency on *NF‐YA1* at this particular stage after *M*.* loti* infection, indicated by the somewhat attenuated response in *nf‐ya1‐2* (Fig. 4). These observations predicted that in addition to *NF‐YA1*, other regulators are likely to partake in mediating *STY* gene expression, which was later confirmed (see below).

In comparison to uninoculated roots, levels of all seven *STY* mRNAs described were also significantly upregulated at subsequent 12 and 21 dai time points. Conversely, *STY4* did not respond significantly and *STY6* mRNA showed decreased levels upon *M*.* loti* infection (Fig. 4).

These observations indicated that at least seven *L*.* japonicus STY* genes were regulated during nodule development, although all nine genes could be pertinent, based on histochemical data. Similarly, *in silico* analysis of *M*.* truncatula* laser caption microscopy/RNA sequencing (LCM‐RNA‐seq) expression data (Roux *et al*., 2014) showed that seven out of eight *MtSTY* genes had significantly higher mRNA levels in mature nodules than in uninoculated roots, primarily in the meristematic zone, with *MtSTY2* and *MtSTY9* displaying the highest fold upregulation (Table S3a). Importantly, upregulated levels of at least six *MtSTY* mRNAs (*MtSTY2*, *3*, *4*, *7*, *8* and *9*), expressed early on upon *S*.* meliloti* infection (Larrainzar *et al*., 2015), were dependent on *MtNF‐YA1* (Table S3b), indicating that the relevant *NF‐YA1*/*STY* regulatory modules operate in both determinate and indeterminate nodules.

### *Lotus japonicus* *sty* mutants

To assess the functional relevance of *STY* genes during root nodule development, mutant *L*.* japonicus* lines carrying *LORE1* retrotransposon insertions in exonic regions were identified for all but the *STY3* gene (Table S4) (https://lotus.au.dk/) (Malolepszy *et al*., 2016; Mun *et al*., 2016). A TILLING approach (Perry *et al*., 2009) was therefore used to identify deleterious mutations at the *STY3* locus. Two identified alleles, *sty3‐1* and *sty3‐9*, carried independent single nucleotide substitutions (C187 to T and C493 to T, respectively) that were predicted to result in premature translation termination (STOP) codons (Fig. S4). The line homozygous for the *sty3‐9* allele was used in subsequent analyses.

Efforts to characterize the impact of individual *sty* mutations on nodulation were focused on *STY1*, *2*, *3*, *7* and *8*, which had upregulated expression 4 d after *M*.* loti* infection (Fig. 4).

### *sty* mutants show weak symbiotic phenotypes

At 7 dai, there were no significant differences in epidermal IT (eIT) formation between wild‐type and the *sty* mutants (Fig. S5a). However, while forming a near wild‐type number of nodules, several *sty* lines had significantly fewer primordia, suggesting gene relevance, particularly where two alleles show this characteristic (e.g. *sty1* and *sty7* loci) (Fig. S5b). Shoot and root weight, used as measures of plant performance, were diminished in most *sty* mutants (Fig. S6a,b), but a simple correlation between plant growth under nonsymbiotic conditions and the number of NP could not be fully supported.

**Fig. 5:**
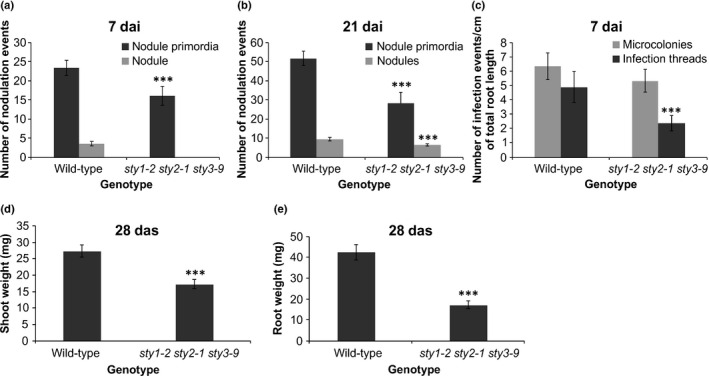
*Lotus japonicus sty1‐2 sty2‐1 sty3‐9* triple mutant has significant symbiotic and nonsymbiotic defects. (a, b) Nodulation events (nodule primordia and nodules) were scored in wild‐type and the triple mutant at 7 (a) and 21 d after inoculation (dai) (b) with *Mesorhizobium loti*. (c) The frequency of infection events (microcolonies and epidermal infection threads) is given. (d, e) Nonsymbiotic phenotypes, including shoot weight (d) and root weight (e) of plants grown under sterile conditions, in the absence of *M*.* loti*, were evaluated 28 d after sowing (das). Twenty and 30 individuals per genotype were analysed for symbiotic and nonsymbiotic phenotypes, respectively. The scores represent means ± 95% confidence intervals for three biological replicates. Asterisks represent a significant difference from wild‐type for a given category (Student’s *t*‐test: ***, *P* < 0.001).

To assess the redundancy of *STY* gene function, a triple *sty* mutant was developed. When evaluated at 21 dai, *sty1‐2 sty2‐1 sty3‐9* had significantly fewer NP, nodules (Fig. 5a) and eITs, while microcolonies (MCs) were unaffected (Fig. 5b). Under nonsymbiotic conditions, 28 d after sowing (das), other developmental failings, including diminished shoot and root growth were apparent (Fig. 5c,d). Given these effects, which confounded the interpretation of the nodulation results, a dominant negative approach, targeting *STY* genes in nodules, was implemented in fully transgenic *L*.* japonicus* plants, extending our earlier observations in the hairy root system (Hossain *et al*., 2016).

### STY3::SRDX blocks infection thread and nodule formation

As *STY3* expression was relatively high in comparison to other *STY* genes and entirely dependent on *NF‐YA1* at 4 dai with *M*.* loti* (Fig. 4), the *LjNF‐YA1_Pro_:STY3::SRDX* chimeric construct (Hossain *et al*., 2016) was used in the dominant negative approach. As documented for several transcription factors (Hiratsu *et al*., 2003), the 12‐amino‐acid‐long Ethylene‐Responsive Element‐Binding Factor (ERF)‐associated amphiphilic repression (SRDX) domain was expected to convert STY3 into a dominant transcriptional repressor.

**Fig. 6:**
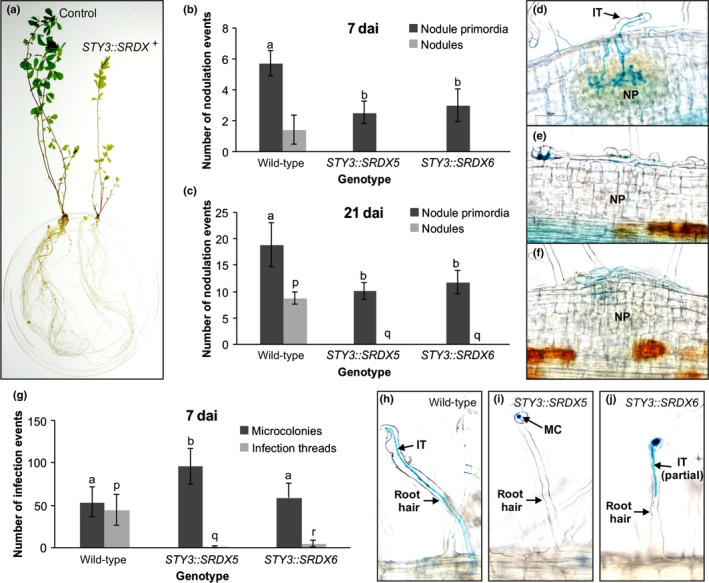
Transgenic *Lotus japonicus* plants carrying the *LjNF‐YA1_Pro_:STY3::SRDX* construct do not form nodules. (a) Images, taken 28 d after inoculation (dai) with *Mesorhizobium loti*, of representative, fully transgenic T0 *L*.* japonicus* plants carrying either empty vector (control), or the same vector containing the *LjNF‐YA1_Pro_:STY3::SRDX* transgene (*STY3::SRDX* ^+^). (b, c) Scores of nodulation events at 7 (b) and 21 dai (c) with *M*.* loti*, from two independent *L*.* japonicus* T1 populations (*STY3::SRDX5* and *STY3::SRDX6*) segregating the *LjNF‐YA1_Pro_:STY3::SRDX* transgene. Note that ‘wild‐type’ denotes T1 segregants without the transgene. (d–f) Representative images of wild‐type nodule primordia (NP) (d) and those formed by transgenic plants carrying the *LjNF‐YA1_Pro_:STY3::SRDX* transgene (e, f). The image in (e) shows a typical, small NP event while panel (f) represents an infrequent, larger NP that forms a visible bump at the root surface. (g) Scores of infection events (i.e. microcolonies and epidermal infection threads) in *L*.* japonicus* T1 plants that either lack (wild‐type) or carry the *LjNF‐YA1_Pro_:STY3::SRDX* transgene. The scores represent means ± 95% confidence intervals. Statistical groupings, reflected by the same letters, have been determined separately for each of the two infection event categories using one‐way ANOVA with Tukey’s honestly significant difference *post hoc* test. (h–j) Representative images of epidermal infection events in the wild‐type (h) and the transgene‐containing (*STY3::SRDX5*) T1 plants (i, j) are shown. MC, microcolony; IT, epidermal infection thread; IT (partial), IT that was terminated within the root hair shaft.

In contrast to profusely nodulated control plants that lacked the construct, 10 independent T0 plants carrying *LjNF‐YA1_Pro_:STY3::SRDX* (*STY3::SRDX* ^+^) formed no visible nodules (Fig. 6a). The remaining, *STY3::SRDX‐*negative (*STY3::SRDX* ^‐^) plants formed wild‐type nodules (Table S5). Detailed evaluation of *STY3::SRDX* ^+^ T1 progeny, at 7 and 21 dai, showed that while unable to develop mature nodules, NP were initiated (Fig. 6b,c), albeit significantly less frequently than in control, *STY3::SRDX* ^‐^ roots (Fig. 6b–f). Despite the *STY3::SRDX* ^+^ plants having many MCs, more than the wild‐type in the case of *STY3::SRDX5*, very few eITs formed (Fig. 6g) and most terminated prematurely within root hairs (Fig. 6h–j). Together, these data indicated an essential role for *STY*s during both eIT formation and nodule organogenesis.

### *STY*s are required to regulate *YUCCA1* and *YUCCA11* expression during symbiosis

Downstream targets of *L*.* japonicus NF‐YA1*/*STY*‐dependent regulation were identified via a targeted approach. Focusing on *YUCCA* genes was deemed relevant given the importance of auxin signalling for both symbiotic infection and nodule formation (Suzaki *et al*., 2012, 2013; Breakspear *et al*., 2014; Kohlen *et al*., 2018) and the postulated role of *NF‐YA1* in regulating the relevant auxin maxima during symbiosis (Hossain *et al*., 2016). *YUCCA* genes encode flavin monooxygenase‐like enzymes and some, including *Arabidopsis* YUCCA4 and YUCCA8, were shown to mediate the rate‐limiting step in tryptophan‐dependent auxin biosynthesis and to be regulated by STY1 (Eklund *et al*., 2010a).

Using *Arabidopsis* YUCCA protein sequences as blast queries (Cheng *et al*., 2006; Zhao, 2018), 21 YUCCA‐like proteins were predicted to be encoded by the *L*.* japonicus* genome, including those represented by only partial sequences (Fig. S7).

An *in silico* analysis of RNA‐seq data (BioProject ID PRJNA630938) showed that at 4 dai with *M*.* loti* the steady‐state level of only *Lj4g3v3081700* (*YUCCA11*) mRNA was significantly (FDR < 0.05) upregulated in *L*.* japonicus* roots (Table S6). A phylogenetic tree constructed with the corresponding protein sequences revealed that *L*.* japonicus* YUCCA11 (LjYUCCA11) had the highest primary sequence homology to *M*.* truncatula* MtrunA17Chr6g0485621/MtYUC2 (Fig. S8), shown previously to respond to *S*.* meliloti* infection (Schiessl *et al*., 2019). Interestingly, three other *Medicago YUCCA* genes, namely *MtrunA17Chr7g0262591* (*Medtr7g09933*0/*MtYUC8*), *MtrunA17Chr7g0262471* (*Medtr7g099160*) and *MtrunA17Chr3g0139441* (*Medtr3g109520*/*MtYUC1*), were upregulated in response to rhizobial inoculation or NF application (Larrainzar *et al*., 2015; Schiessl *et al*., 2019). The predicted protein products of the first two *M*.* truncatula* genes had the highest homology to Lj1g3v4528740.1 (LjYUCCA1) while the third had the highest homology to Lj1g3v2036560.1 (Fig. S8). Considering both RNA‐seq data and phylogenic relationships, *LjYUCCA1* and *LjYUCCA11* genes were chosen for subsequent analyses.

**Fig. 7:**
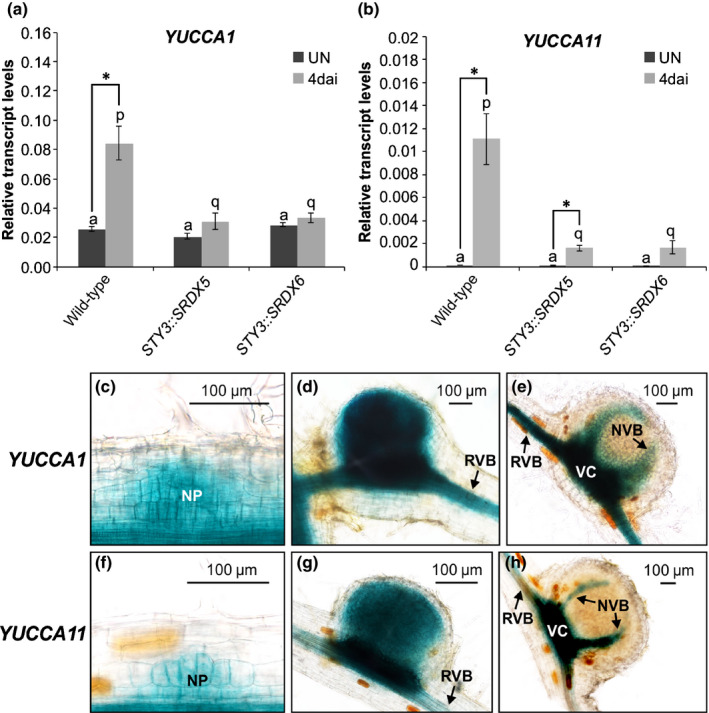
*Lotus japonicus YUCCA1* and *YUCCA11* expression associates with nodule development and is dependent upon *STY*s. (a, b) Steady‐state levels of *YUCCA1* (a) and *YUCCA11* (b) mRNAs were determined by quantitative reverse transcription polymerase chain reaction in uninoculated (UN) *L*.* japonicus* roots of the wild‐type genotype and the corresponding roots from T0 transgenic plants, propagated from cuttings of two independent T0 lines (*STY3::SRDX5* and *STY3::SRDX6*) carrying the *STY3‐SRDX* transgene. These were compared with equivalent root samples collected 4 d after inoculation (dai) with *Mesorhizobium loti*. The mean ± SE is given for three biological replicates. Statistical groupings across different genotypes, reflected by the same letters, have been determined separately for *M*.* loti* inoculated and uninoculated samples using one‐way ANOVA with Tukey’s honestly significant difference *post hoc* test. Asterisks (*) denote significant differences between uninoculated and inoculated samples (Student’s *t*‐test: *, *P* < 0.05). (c–h) The β‐glucuronidase (GUS) reporter activity (in blue) as driven by *YUCCA1* (c–e) and *YUCCA11* (f–h) promoters. Representative images of nodule primordia (c, f) and small (d, g) and fully mature nodules (e, h) are shown. The images were captured at 10 dai with *M*.* loti*. NP, nodule primordium; NVB, nodule vascular bundle; RVB, root vascular bundle; VC, vascular cambium.

*YUCCA1* mRNA, while detectable in uninoculated *L*.* japonicus* roots, was significantly increased upon *M*.* loti* infection (Fig. 7a). By contrast, *YUCCA11* mRNA could be detected only in *M*.* loti*‐inoculated roots (Fig. 7b). Importantly, the responsiveness of the two genes was significantly attenuated in *STY3::SRDX5* and *STY3::SRDX6* roots (Fig. 7a,b).

The *YUCCA1* promoter showed activity along the entire root vasculature and also in dividing cortical cells of NP (Fig. 7c,e). In developing nodules, this activity appeared to be present in all centrally located cells, only to be confined later to the nodule vasculature in mature nodules (Fig. 7d,e). The activity of the *YUCCA11* promoter, though similar, was confined to regions of the root associated with nodules, including vasculature at the place of nodule emergence (Fig. 7f,h).

### NF‐YA1 regulates expression of YUCCA1 and YUCCA11

Because responsiveness of *YUCCA1* and *YUCCA11* upon *M*.* loti* infection required *STY‐*dependent functions and *STY*s were regulated by *NF‐YA1*, the two *YUCCA* genes should also be subjected to the *NF‐YA1*‐dependent regulation.

**Fig. 8:**
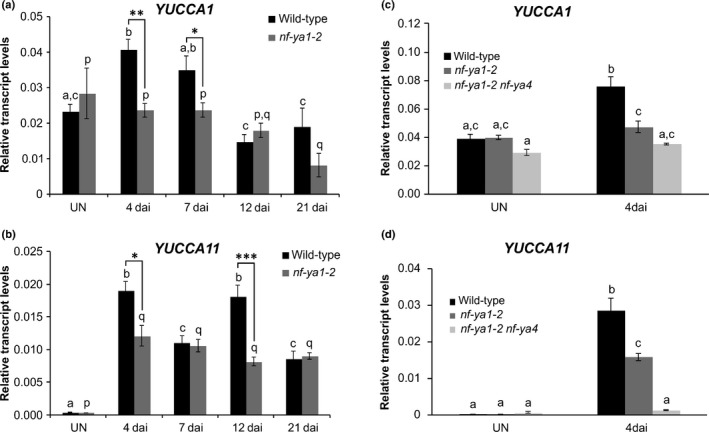
*Lotus japonicus NF‐YA1* and *NF‐YA4* work partially redundantly to regulate *YUCCA11* gene expression. (a–d) Quantitative reverse transcription polymerase chain reaction data showing steady‐state levels of *YUCCA1* (a, c) and *YUCCA11* (b, d) mRNAs in roots of *L*.* japonicus* wild‐type (black), *nf‐ya1‐2* (dark grey) and the *nf‐ya1‐2 nf‐ya4* double mutant (light grey). Root samples from the uninoculated (UN) control and those collected at various time points (d) after inoculation (dai) with *Mesorhizobium loti* were analysed. In all graphs, the mean ± SE is given for three biological replicates. Small letters denote significant differences in transcript abundance between time points, as determined separately for each genotype by one‐way ANOVA with Tukey’s honestly significant difference *post hoc* test. Student’s *t*‐test was used to carry out pairwise comparisons between wild‐type and *nf‐ya1‐2* in (a) and (b). Asterisks denote significant differences in pairwise comparisons (*, *P* < 0.05; **, *P* < 0.01; ***, *P* < 0.001).

For *YUCCA1* this was most apparent at 4 dai, where the mRNA level was significantly upregulated above control, uninoculated roots and this was entirely *NF‐YA1*‐dependent (Fig. 8a). By contrast, levels of *YUCCA11* mRNA remained high in *M*.* loti*‐inoculated roots at all time points analysed. Interestingly, reaching peak levels at 4 and 12 dai required *NF‐YA1*, while maintaining moderately upregulated levels at 7 and 21 dai time points was *NF‐YA1*‐independent (Fig. 8b).

### *NF‐YA1* and *NF‐YA4* function partially redundantly to regulate *YUCCA11* and *STY*s

It was surmised that another, partially redundantly operating *NF‐YA* could be responsible for the observed incomplete *NF‐YA1* dependency of the *YUCCA11* gene expression. As *L*.* japonicus NF‐YA4* is predicted to be the closest paralogue of *NF‐YA1* (Hossain *et al*., 2016), we tested whether it is pertinent to *YUCCA11* and perhaps also to *YUCCA1* expression.

The *nf‐ya4* mutant allele was derived from a *L*.* japonicus* line carrying the *LORE1* insertion in the first exon of the *NF‐YA4* locus (Table S7). At 4 dai with *M*.* loti*, the steady‐state level of *YUCCA1* mRNA was significantly upregulated in wild‐type but not in *nf‐ya1‐2* mutant roots, and absence of functional *NF‐YA4* in *nf‐ya1‐2 nf‐ya4* had no additional significant impact (Fig. 8c). By contrast, strong upregulation of *YUCCA11* mRNA, in the *M*.* loti*‐inoculated wild‐type, was significantly attenuated in *nf‐ya1‐*2 and further diminished to the background, uninoculated level, in *nf‐ya1‐2 nf‐ya4* roots, (Fig. 8d).

**Fig. 9:**
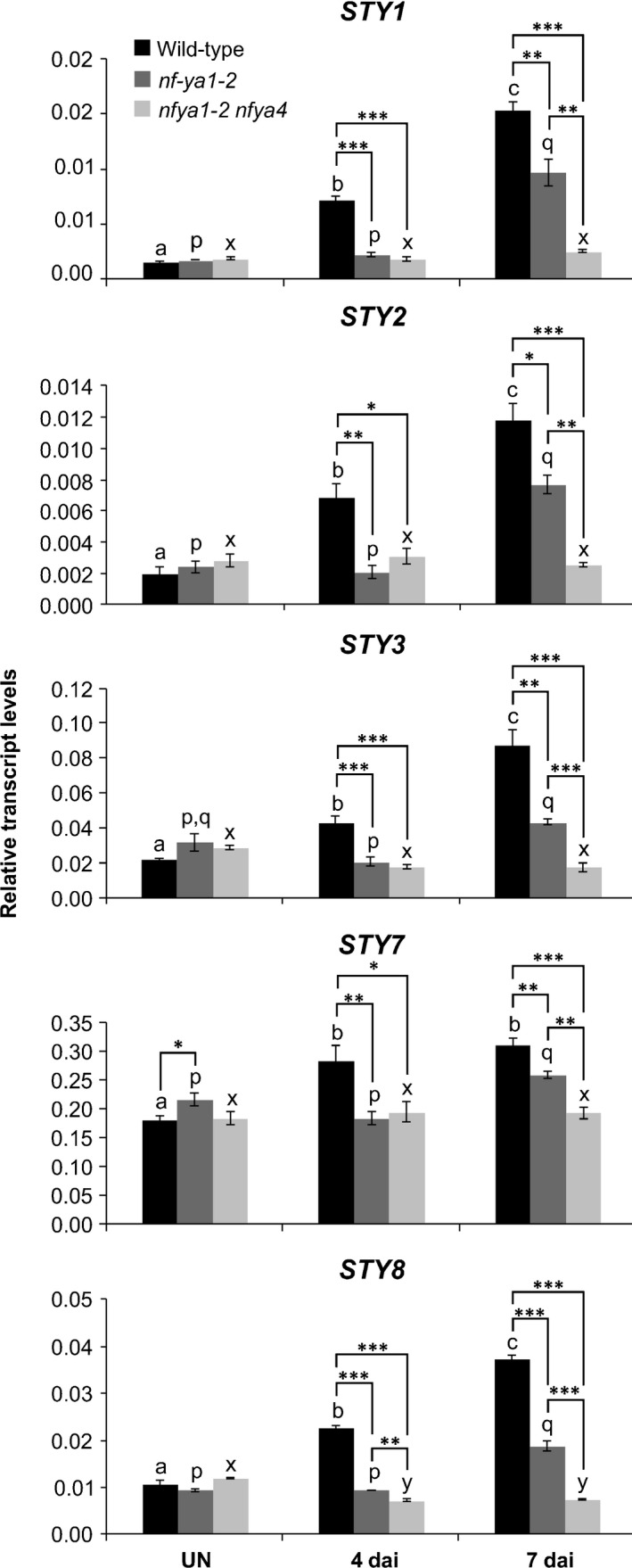
*Lotus japonicus NF‐YA1* and *NF‐YA4* work partially redundantly to regulate expression of *STY* genes. The steady‐state levels of *L*.* japonicus STY* mRNAs in control, uninoculated (UN) roots of *L*.* japonicus* wild‐type (black), *nf‐ya1‐2* (dark grey) and *nf‐ya1‐2 nf‐ya4* mutants (light grey) and in corresponding root samples collected at 4 and 7 d after inoculation (dai) with *Mesorhizobium loti*. The mean ± SE is given for four to five biological replicates. Statistical groupings across different time points, reflected by the same letters, have been determined separately for each genotype using one‐way ANOVA with Tukey’s honestly significant difference *post hoc* test (*P* < 0.05). Dunnett’s test was used to carry out pairwise comparisons between wild‐type, *nf‐ya1‐2* and *nf‐ya1‐2 nf‐ya4* mutants at each time point (*, *P* < 0.05; **, *P* < 0.01; ***, *P* < 0.001).

We then asked whether this apparent functional redundancy between *NF‐YA1* and *NF‐YA4* could also explain the *NF‐YA1*‐independent regulation of *STY* gene expression, as observed at 7 dai with *M*.* loti* (see Fig. 4) and we found this to be the case (Fig. 9).

### *NF‐YA4* acts partially redundantly with *NF‐YA1* to regulate symbiosis

**Fig. 10:**
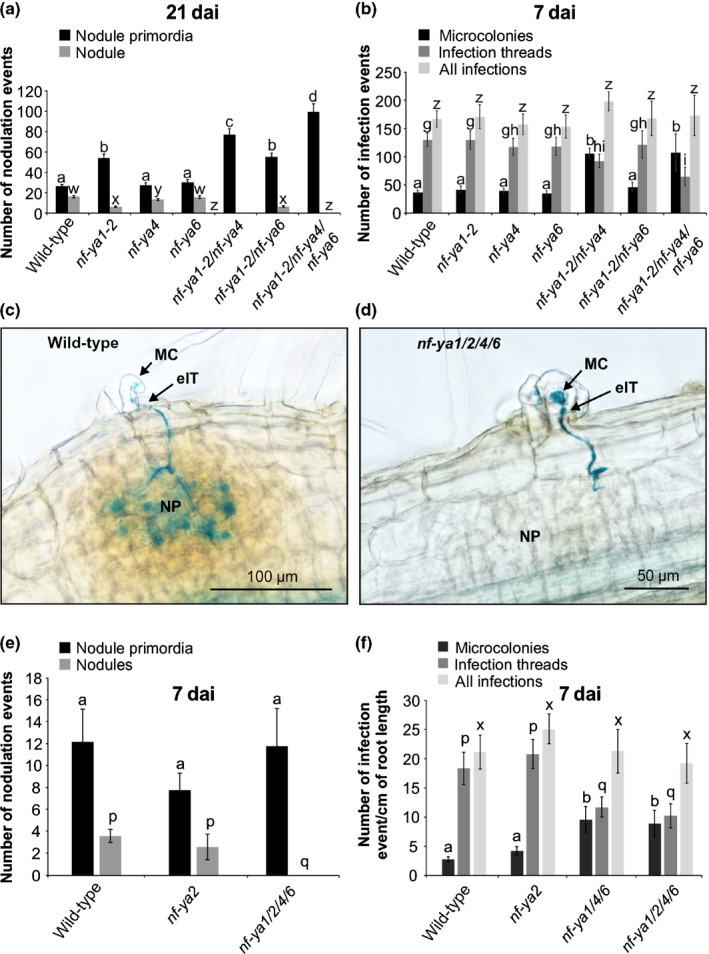
*Lotus japonicus NF‐YAs* function partially redundantly to regulate nodule organogenesis and *Mesorhizobium loti* infection. (a, b, e, f) Scores of nodulation events (nodule primordia and nodules) at 21 d after inoculation (dai) (a) and the number of nodulation (e) and infection events (b, f) (at 7 dai with *M*.* loti* strain NZP2235 carrying the *hemA:LacZ* reporter cassette for the indicated genotypes. Means ± 95% confidence intervals are presented for 15–54 individuals per genotype and statistical groupings, reflected by the same letters, have been determined separately for each category using one‐way ANOVA with Tukey’s honestly significant difference *post hoc* test (*P* < 0.05). (c, d) Typical wild‐type (c) and *nf‐ya1/2/4/6* mutant (d) nodule primordia (NP). MC, microcolony; eIT, epidermal infection thread.

Consistent with its impact on *STY* and *YUCCA* gene expression, *nf‐ya4* potentiated the symbiotic defect of *nf‐ya1‐2*. At 21 dai with *M*.* loti*, *nf‐ya1‐2* roots developed a few nodules (Fig. 10a), while the *nf‐ya1‐2 nf‐ya4* double mutant formed only small NP, developmentally arrested at a stage comparable to those formed by *LjNF‐YA1_Pro_:STY3::SRDX* transgenic plants (Fig. 6e,f). However, unlike the transgenic plants, the number of NP in *nf‐ya1‐2 nf‐ya4* was elevated compared with wild‐type and *nf‐ya1‐2* single mutant plants (Fig. 10a). At 7 dai with *M*.* loti*, *nf‐ya1‐2 nf‐ya4* also showed a weak but significant infection defect, forming fewer eITs and more MCs than the wild‐type (Fig. 10b).

Our RNA‐seq data (BioProject ID PRJNA630938) from uninoculated *L*.* japonicus* roots collected 11 das showed that the *NF‐YA4* mRNA was the most abundant among all root *NF‐YA* mRNA species, followed by *NF‐YA2 *> *6* > *5 *> *7* > *8 *> *3* and > *1* (Table S8). We therefore tested symbiotic phenotypes of higher‐order mutants, *nf‐ya1‐2 nf‐ya4 nf‐ya6* and *nf‐ya1‐2 nf‐ya2 nf‐ya4 nf‐ya6*. *Lotus* *japonicus* lines carrying exonic *LORE1* insertions at the *NF‐YA2* and *NF‐YA6* loci were used to develop these mutant lines (Table S7). Like *nf‐ya1‐2 nf‐ya4*, the triple and quadruple *nf‐ya* mutants did not develop nodules but retained the capacity to make small NP (Fig. 10c,e). At 21 dai, these were significantly more abundant than in corresponding wild‐type roots (Figs 10a, S9) and this was in spite of somewhat diminished root growth of the mutant lines. By contrast, the number of eITs was reduced by *c*. 50%, while the number of MCs was significantly increased in both triple and quadruple mutants (Fig. 10b,f). The direct comparison between the *nf‐ya1/4/*6 and *nf‐ya1/2/4/6* mutant lines showed no significant difference with respect to number and category of infection events (Fig. 10f).

### Early auxin signalling remains unaffected in the *nf‐ya1 nf‐ya4* double mutant

**Fig. 11:**
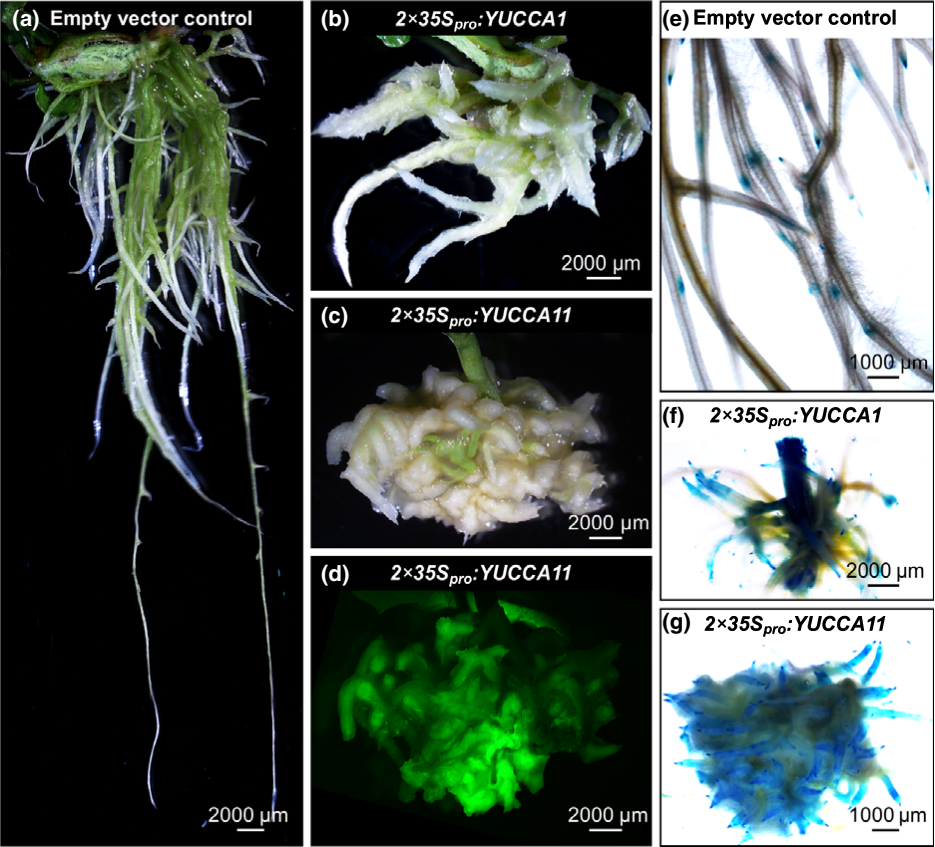
Ectopic expression of *YUCCA1* and *YUCCA11* results in auxin overproduction root phenotypes. (a–c) Representative images of hairy roots generated on transgenic *Lotus japonicus* shoots, carrying the *DR5:GUS* auxin reporter, using *Agrobacterium rhizogenes* carrying empty pKGWD,0 vector (a), or the same vector containing either the *YUCCA1* mRNA sequence (b) or the *YUCCA11* genomic fragment (including all exons, introns and the 3′UTR) (c) under the control of 2x *CaMV 35S* promoter (2x35S_pro_). (d) Example of the green fluorescent protein (GFP) fluorescence for the specimen shown in (c), used as proof of successful transformation. Panels (e–g) show activity of the *DR5:GUS* reporter, indicated in blue, in the corresponding hairy roots. Note that independent hairy root specimens were used for live imaging (a–d) and the histochemical analysis of β‐glucuronidase (GUS) activity (e–g).

Having determined that *NF‐YA*/*STY* module‐dependent signalling regulates *YUCCA1* and *YUCCA11* in the context of symbiosis, and that the relevant members of the three gene families shared nodule expression domains, we asked whether this contributes to auxin signalling. The corresponding *YUCCA1* cDNA and *YUCCA11* genomic sequences were therefore expressed under the control of the *CaMV* 2x35S constitutive promoter in hairy roots induced on transgenic *L*.* japonicus* shoots carrying the *DR5*:*GUS* auxin reporter (Ulmasov *et al*., 1997; Liao *et al*., 2015). Control hairy roots had typical elongated morphology, with the reporter activity confined to lateral root initiation sites and root apical regions (Fig. 11a,e). By contrast, 2x35S*_Pro_*:*YUCCA1* and 2x35S*_Pro_*:*YUCCA11*‐transformed hairy roots had a *super‐root*‐like morphology (Boerjan *et al*., 1995), with 2x35S*_Pro_*:*YUCCA11* having the most pronounced impact on phenotype (Fig. 11b,d). Their short, thick and highly branched architecture was associated with ectopic *DR5:GUS* activity, which extended well beyond apical root regions, indicative of enhanced auxin activity (Fig. 11f,g). These observations suggested that *YUCCA1* and *YUCCA11* participate in regulation of auxin homeostasis, which was further tested.

**Fig. 12:**
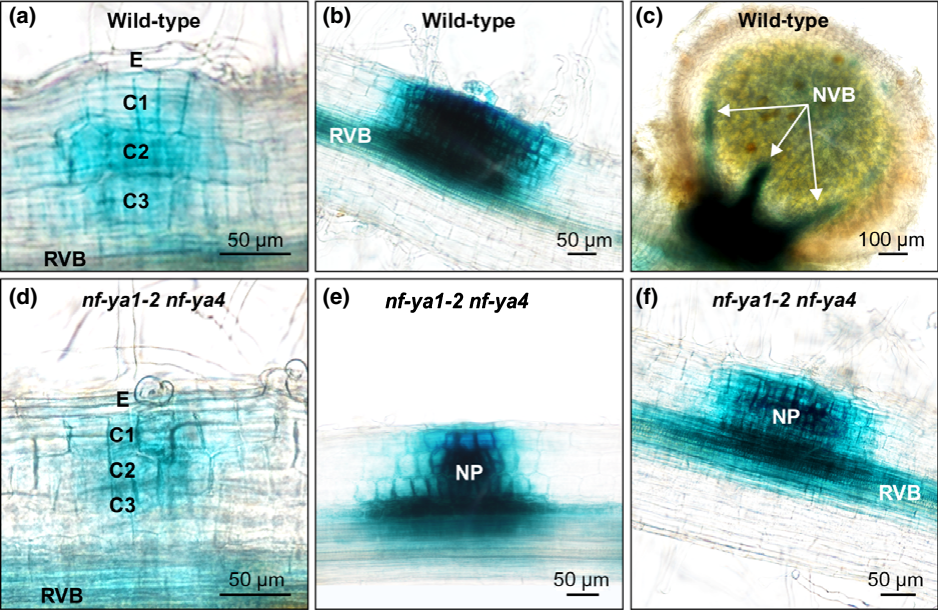
Localization of auxin responses during nodule development in *Lotus japonicus* wild‐type and *nf‐ya1‐2 nf‐ya4*. Representative images of the *DR5:GUS* reporter activity, indicated in blue, during different stages of nodule formation in *L*.* japonicus* wild‐type (upper row) and the *nf‐ya1‐2 nf‐ya4* double mutant (lower row). (a–f) The specimens were collected and imaged at 14 (a, b, d, e) and 21 (c, f) d after inoculation (dai) with *Mesorhizobium loti*. Note that nodule development is blocked in *nf‐ya1‐2 nf‐ya4* such that at 21 dai only nodule primordia (NP) are present (f). RVB, root vascular bundle; NVB, nodule vascular bundle; E, epidermis; C1, C2 and C3, consecutive root cortical cell layers.

The *DR5:GUS* reporter activity in wild‐type *M*.* loti*‐inoculated *L*.* japonicus* plants was associated with both early and late symbiotic events, initially observed in conjunction with early cell divisions in subepidermal and two subtending cortical cell layers (Fig. 12a) and slightly later during development, across all cell layers of small NP (Fig. 12b). Notably, similar early *DR5:GUS* activity patterns were also present in the *nf‐ya1‐2 nf‐ya4* double mutant (Fig. 12d,e). At 21 dai with *M*.* loti*, wild‐type plants had mature nodules with *DR5:GUS* activity confined to the base and vasculature (Fig. 12c), reminiscent of the *L*.* japonicus NF‐YA1* and *STY* gene expression (Fig. 2; Hossain *et al*., 2016). By contrast, in the double mutant, which is unable to make nodules, the reporter gene activity continued to associate with small NP (Fig. 12f). We attempted to rescue the *nf‐ya1 nf‐ya4* mutant nodulation phenotype in transgenic hairy root experiments using a chimeric *NF‐YA1*_*Pro*_:*YUCCA11* expression module, but this was unsuccessful as no emerged nodules were present on transformed hairy roots. It is likely that in spite of a similar spatiotemporal expression patterns during nodule development, the strong *NF‐YA1* promoter cannot functionally mimic the weak *YUCCA11* promoter, resulting in lack of complementation. *NF‐YA1* may also regulate other necessary processes in parallel to *YUCCA* genes.

## Discussion

We have demonstrated previously that *NF‐YA1* regulates expression of three members of the *L*.* japonicus SHI/STY* gene family (Hossain *et al*., 2016). This was extended by showing that the activity of all nine *L*.* japonicus STY* genes is associated with nodule development, and that seven are *NF‐YA1‐*dependent. Similarly, expression of seven *M*.* truncatula STY* genes was upregulated in nodules and at least six of them showed *MtNF‐YA1* dependency. *Arabidopsis LRP1* (Smith & Fedoroff, 1995), *STY2* (Kuusk *et al*., 2002), *SRS5* (Kuusk *et al*., 2006) and *STY1* (Eklund *et al*., 2011) shared identical or highly overlapping expression domains. This was also true for promoters of the *L*.* japonicus STY* genes, implying their functional redundancy in mediating root and nodule development. Importantly, *STY* gene activities in mature nodules associated with vascular bundles and meristematic regions in *L*.* japonicus* and *M*.* truncatula* nodules, respectively, probably reflecting areas of persisting cell cycle activities in these two developmentally distinct organs.

A remarkable feature of *SHI/STY* genes is their functional synergism and redundancy. Phenotypic features of single and higher‐order *Arabidopsi*s mutants indicate that members of the *SHI*/*STY* family act in a dosage‐dependent manner to regulate carpel and leaf formation (Kuusk *et al*., 2006). Unlike leaves and carpels, however, nodules are dispensable plant organs. Therefore, finding that expression of nine *L*.* japonicus* and at least six *M*.* truncatula STY* genes associates with nodule development was surprising. As the *STY*s were expressed at a relatively low level in *L*.* japonicus* roots, a dosage‐dependency requirement for accurate SHI/STY functioning during nodule formation could be a relevant factor. On the other hand, selective constraints for maintaining such a large number of *SHI/STY* genes in the symbiotic programme could be owing to its initial, extensive overlap with the lateral root formation programme (Schiessl *et al*., 2019; Soyano *et al*., 2019).

### *STY*s regulate rhizobial infection and early nodule primordia formation events

Both the *sty1/2/3* triple mutant and the *LjNF‐YA1_Pro_:STY3::SRDX* transgenic plants had significantly fewer eITs than did the wild‐type, despite an unchanged or even increased number of MCs, as in the *STY3::SRDX5* background (Figs 5, 6). Auxin has been shown to play an important role in mediating symbiotic infection in root hairs (Breakspear *et al*., 2014; Nadzieja *et al*., 2018). Hence, *STY*s and *YUCCA*s may contribute to the mechanism that regulates auxin biosynthesis during IT formation. This notion is consistent with the observation that the soybean *GmYUC2a* gene, while contributing to local auxin biosynthesis, was essential for both *Bradyrhizobium diazoefficiens* infection and nodule organogenesis (Wang *et al*., 2019).

*STY*s may also partake in the regulation of early NP formation. Transgenic *LjNF‐YA1_Pro_:STY3::SRDX* plants formed significantly fewer (*c*. 50%) early NP compared with the wild‐type. A similar negative effect was observed in *sty1/2/3*, suggesting that *STY*s are indeed required in this developmental context. If so, this is most likely independent of *NF‐YA1/NF‐YA4*.

### *Lotus japonicus NF‐YA*s, unlikely participants of early nodule primordia formation events

**Fig. 13:**
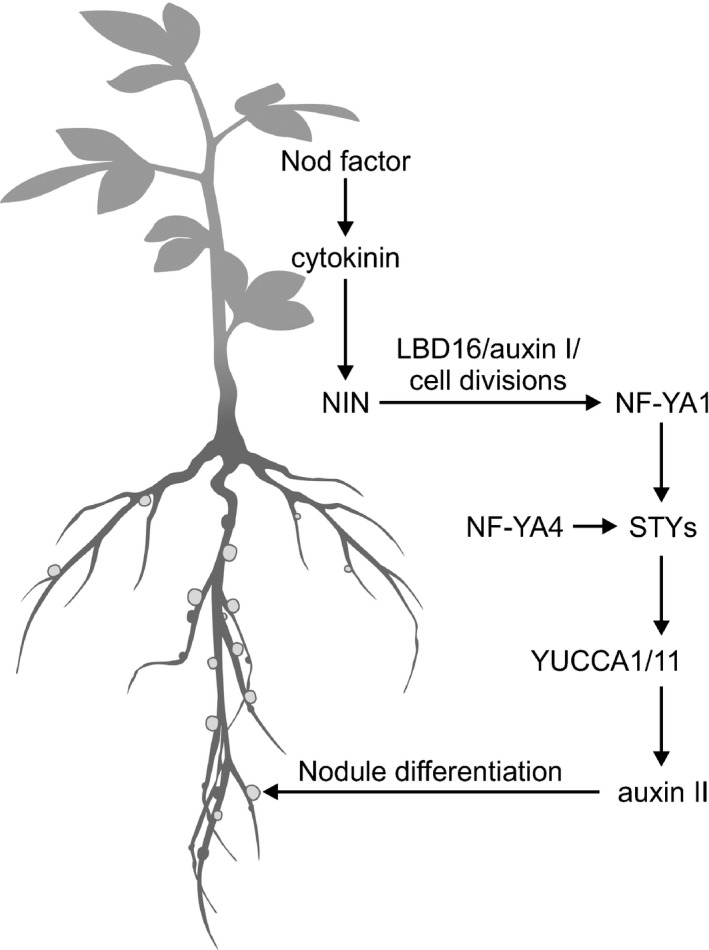
A working model for the *NF‐YA*‐dependent regulation of nodule organogenesis in *Lotus japonicus*. The *Mesorhizobium loti*‐derived nodulation (Nod) factor regulates expression of *NF‐YA1* in a cytokinin‐ and *NODULE INCEPTION* (*NIN*)‐dependent manner. Acting as a presumed subunit of a heterotrimeric NF‐Y complex, NF‐YA1, along with NF‐YA4, partakes in the activation of *STY* gene expression in young nodule primordia, which in turn regulates the activity of *YUCCA1* and *YUCCA11* genes. The latter two genes are predicted to encode flavin monooxygenases, which contribute to local auxin signalling (auxin II) that mediates differentiation of mature nodules, downstream from the initial cell divisions that form nodule primordium in association with the first auxin peak (auxin I).

Both *nf‐ya1‐2* and *nf‐ya1‐2 nf‐ya4* formed significantly more early NP than did the wild‐type, yet the additive effect of the *nf‐ya4* mutation totally blocked nodule emergence in the double mutant (Fig. 10). Formation of eITs was also affected to some degree in *nf‐ya1 nf‐ya4*. However, the total number of infections (i.e. number of MCs and eITs) was similar to the wild‐type, indicating that root susceptibilty to *M*.* loti* infection was unchanged in the absence of *NF‐YA1* and *NF‐YA4*. Higher‐order *nf‐ya* mutants were tested but no additional significant effects were found, and at 21 dai the capacity of *nf‐ya1/2/4/6* to form early NP was enhanced relative to the wild‐type. Additional *NF‐YA* genes are expressed at relatively low levels in *L*.* japonicus* roots (Table S8). We cannot, therefore, entirely rule out the possibility that higher redundancy, beyond the *NF‐YA1/2/4/6* level, accounts for the sustained early NP formation in the quadruple mutant. However, we consider this rather unlikely because in the presence of *nf‐ya1‐2*, the *nf‐ya4* mutation totally blocked the responsiveness of *STY* and *YUCCA* genes to *M*.* loti* infection, but NP were still formed. We therefore favour a model wherein *L*.* japonicus NF‐YA1* is essential only downstream of the initial cortical cell divisions. Together with other partially redundantly operating *NF‐YA*s, such as *NF‐YA4*, *NF‐YA1* probably mediates the nodule emergence stage by regulating *STY*s and *YUCCA*s involved in auxin biosynthesis (Fig. 13). Consistent with this model, early auxin activity, required for or associated with the initial cell divisions (Suzaki *et al*., 2012; Ng & Mathesius, 2018; Kohlen *et al*., 2018), appeared to be wild‐type in *nf‐ya1‐2 nf‐ya4*, indicating that an independent mechanism must mediate this process. Future experiments should test the impact of *YUCCA1* and *YUCCA11* silencing on the nodulation phenotype.

### *Lotus* *japonicus NF‐YA1* mediates local auxin signalling required for nodule emergence

A rapid increase in acropetal auxin transport below the *M*.* loti* infection sites has been documented in *L*.* japonicus* roots, but how this relates to early nodule primodium formation remains unclear (Pacios‐Bras *et al*., 2003; Ng & Mathesius, 2018). An increase in the cellular sensitivity to auxin was also considered in this developmental context, but this assertion awaits further confirmation (Ng & Mathesius, 2018). Finding that *LBD16*/*ASL1*, a member of a plant‐specific transcription factor gene family (Shuai *et al*., 2002), mediates local auxin biosynthesis preceding activation of the cell cycle for NP therefore provides an important insight into at least one of the contributing mechanisms (Schiessl *et al*., 2019).

LOB‐domain proteins regulate developmental and metabolic processes (Shuai *et al*., 2002; Xu *et al*., 2016) and *Arabidopsis LBD16*/*ASL18* was shown to participate in the initiation of various root and root‐like organs by serving as a point of convergence for different priming events (W. Liu *et al*., 2018). This is probably also the role of legume *LBD16*/*ASL18* during nodule formation (Schiessl *et al*., 2019; Soyano *et al*., 2019). Consistent with our results in *L*.* japonicus* and also previous observations (Combier *et al*., 2006; Laloum *et al*., 2014; Laporte *et al*., 2014; Baudin *et al*., 2015), the *M*.* truncatula nf‐ya1‐1* mutation had little impact in this context (Schiessl *et al*., 2019). A fate mapping approach has shown that the apical meristem, typical of indeterminate nodules, specifically originates from divisions in the third root cortical layer (C3). In the *M*.* truncatula nf‐ya1‐1* knockout mutant, NP development was unaffected, while cell divisions in the C3 layer and consequently nodule meristem formation were strongly impaired (Xiao *et al*., 2014).

Clearly, both LBD16 and NF‐YA1 are positive regulators of cell divisions (Schiessl *et al*., 2019; Soyano *et al*., 2019). However, their interaction in *L*.* japonicus*, under innate conditions, is likely to be pertinent only downstream of the initial cell divisions, perhaps during the subsequent cell division maintenance phase (Suzaki *et al*., 2012). Interestingly, different developmental processes apparently require a gradual attenuation of the *LBD16* expression at the patterning stage (W. Liu *et al*., 2018) and based on the published expression data (Schiessl *et al*., 2019), this also seems to be the case during root nodule formation. By contrast, *NF‐YA1*s remain highly expressed and are indispensable for nodule differentiation (Combier *et al*., 2006; Laloum *et al*., 2014; Hossain *et al*., 2016).

We propose that the initial cell divisions and subsequent nodule emergence, encompassing further cell divisions and nodule patterning, are regulated by *NF‐YA1*‐independent and dependent mechanisms, respectively (Fig. 13). Our data lend experimental support for the previously postulated, developmental stage‐specific regulation of determinate and indeterminate nodule formation (Suzaki *et al*., 2012; Xiao *et al*., 2014) while depicting *L*.* japonicus* and *M*.* truncatula NF‐YA1* genes as important nodule emergence stage‐specific regulators of auxin signalling.

## Author contributions

KS, AS and AN conceived the idea and designed the experiments. AS, SZ, JT, TH, AL and LR performed the experiments. SS, AS and AN analysed the gene families. TM, SUA and JS analysed RNA‐seq data and provided bioinformatics and LORE1 mutant support. KS, AS and LR wrote the article.

## Supporting information


**Fig. S1** *Lotus japonicus* STY proteins.
**Fig. S2** The putative RING zinc‐finger domain.
**Fig. S3** Predicted IGGH domain.
**Fig. S4** *Lotus japonicus* mutant *sty* alleles.
**Fig. S5** *Lotus japonicus* single *sty* mutants have only very subtle symbiotic defects.
**Fig. S6** Mutations at most *STY* loci affect nonsymbiotic plant growth.
**Fig. S7** Primary sequence conservation between predicted *Lotus japonicus* YUCCA proteins.
**Fig. S8** Relationship tree between predicted *Lotus japonicus* and *Medicago truncatula* YUCCA proteins.
**Fig. S9** *Lotus japonicus* *NF‐YAs* function partially redundantly.
**Table S1** Primers used in this study.
**Table S2** Expression of four *Lotus japonicus STY* genes is significantly upregulated during the early stages of symbiosis.
**Table S3** Analysis of *Medicago truncatula STY* gene expression.
**Table S4** List of *sty* alleles carrying a LORE1 insertion, as identified from the Lotus Base information portal (https://lotus.au.dk/).
**Table S5** Segregation of the *NF‐YA1_Pro_:STY3::SRDX* transgene in T1 populations, *STY3::SRDX5* and *STY3::SRDX6*, derived from two independent T0 plants.
**Table S6** *YUCCA11* is regulated upon *Mesorhizobium loti* inoculation.
**Table S7** A list of mutant *nf‐ya* alleles used in this study.
**Table S8** Levels of different *NF‐YA* mRNAs in uninoculated *L*. *japonicus* roots.
Please note: Wiley Blackwell are not responsible for the content or functionality of any Supporting Information supplied by the authors. Any queries (other than missing material) should be directed to the New Phytologist Central Office.
