# Supplementary material for: Lotus japonicus Nuclear Factor YA1, a nodule emergence stage‐specific regulator of auxin signalling
Source: New Phytol. 2020 Nov 2;229(3):1535–52. doi: 10.1111/nph.16950 (PMC7984406; doi:10.1111/nph.16950)
Supplement: Supplementary file 1 — Fig. S1 Lotus japonicus STY proteins. Fig. S2 The putative RING zinc‐finger domain. Fig. S3 Predicted IGGH domain. Fig. S4 Lotus japonicus mutant sty alleles. Fig. S5 Lotus japonicus single sty mutants have only very subtle symbiotic defects. Fig. S6 Mutations at most STY loci affect nonsymbiotic plant growth. Fig. S7 Primary sequence conservation between predicted Lotus japonicus YUCCA proteins. Fig. S8 Relationship tree between predicted Lotus japonicus and Medicago truncatula YUCCA proteins. Fig. S9 Lotus japonicus NF‐YAs function partially redundantly. Table S1 Primers used in this study. Table S2 Expression of four Lotus japonicus STY genes is significantly upregulated during the early stages of symbiosis. Table S3 Analysis of Medicago truncatula STY gene expression. Table S4 List of sty alleles carrying a LORE1 insertion, as identified from the Lotus Base information portal (https://lotus.au.dk/). Table S5 Segregation of the NF‐YA1Pro:STY3::SRDX transgene in T1 populations, STY3::SRDX5 and STY3::SRDX6, derived from two independent T0 plants. Table S6 YUCCA11 is regulated upon Mesorhizobium loti inoculation. Table S7 A list of mutant nf‐ya alleles used in this study. Table S8 Levels of different NF‐YA mRNAs in uninoculated L. japonicus roots. Please note: Wiley Blackwell are not responsible for the content or functionality of any Supporting Information supplied by the authors. Any queries (other than missing material) should be directed to the New Phytologist Central Office. [file NPH-229-1535-s001.pdf]

New Phytologist Supporting Information

Article title: *Lotus japonicus Nuclear Factor YA1,* a nodule emergence stage-specific regulator of auxin signalling.

Authors: Arina Shrestha, Sihui Zhong, Jasmine Therrien, Terry Huebert, Shusei Sato, Terry Mun, Stig U. Andersen, Jens Stougaard, Agnes Lepage, Andreas Niebel, Loretta Ross and Krzysztof Szczyglowski*

Article acceptance date: 05 September 2020

The following Supporting Information is available for this article:

Fig. S1. *Lotus japonicus* STY proteins.

Fig. S2. The putative RING zinc-finger domain.

Fig. S3. Predicted IGGH domain.

Fig. S4. *Lotus japonicus* mutant *sty* alleles.

Fig. S5. *Lotus japonicus* single *sty* mutants have only very subtle symbiotic defects.

Fig. S6. Mutations at most *STY* loci affect non-symbiotic plant growth.

Fig. S7. Primary sequence conservation between predicted *Lotus japonicus* YUCCA proteins.

Fig. S8. Relationship tree between predicted *Lotus japonicus* and *Medicago truncatula* YUCCA proteins.

Fig S9. *Lotus japonicus NF-YAs* function partially redundantly

Table S1. Primers used in this study.

Table S2. Expression of four *Lotus japonicus STY* genes is significantly upregulated during early stages of symbiosis.

Table S3. Analysis of *Medicago truncatula STY* gene expression.

Table S4. List of *sty* alleles carrying a LORE1 insertion, as identified from the Lotus Base information portal (https://lotus.au.dk/).

Table S5. Segregation of the *proNF-YA1:STY3::SRDX* transgene in T1 populations, *STY3::SRDX5* and *STY3::SRDX6*, derived from two independent T0 plants.

Table S6. *YUCCA11* is regulated upon *Mesorhizobium loti* inoculation.

Table S7. A list of mutant *nf-ya* alleles used in this study.

Table S8. Levels of different *NF-YA* mRNAs in un-inoculated *L. japonicus* roots.

Fig. S1: *Lotus japonicus* STY proteins.

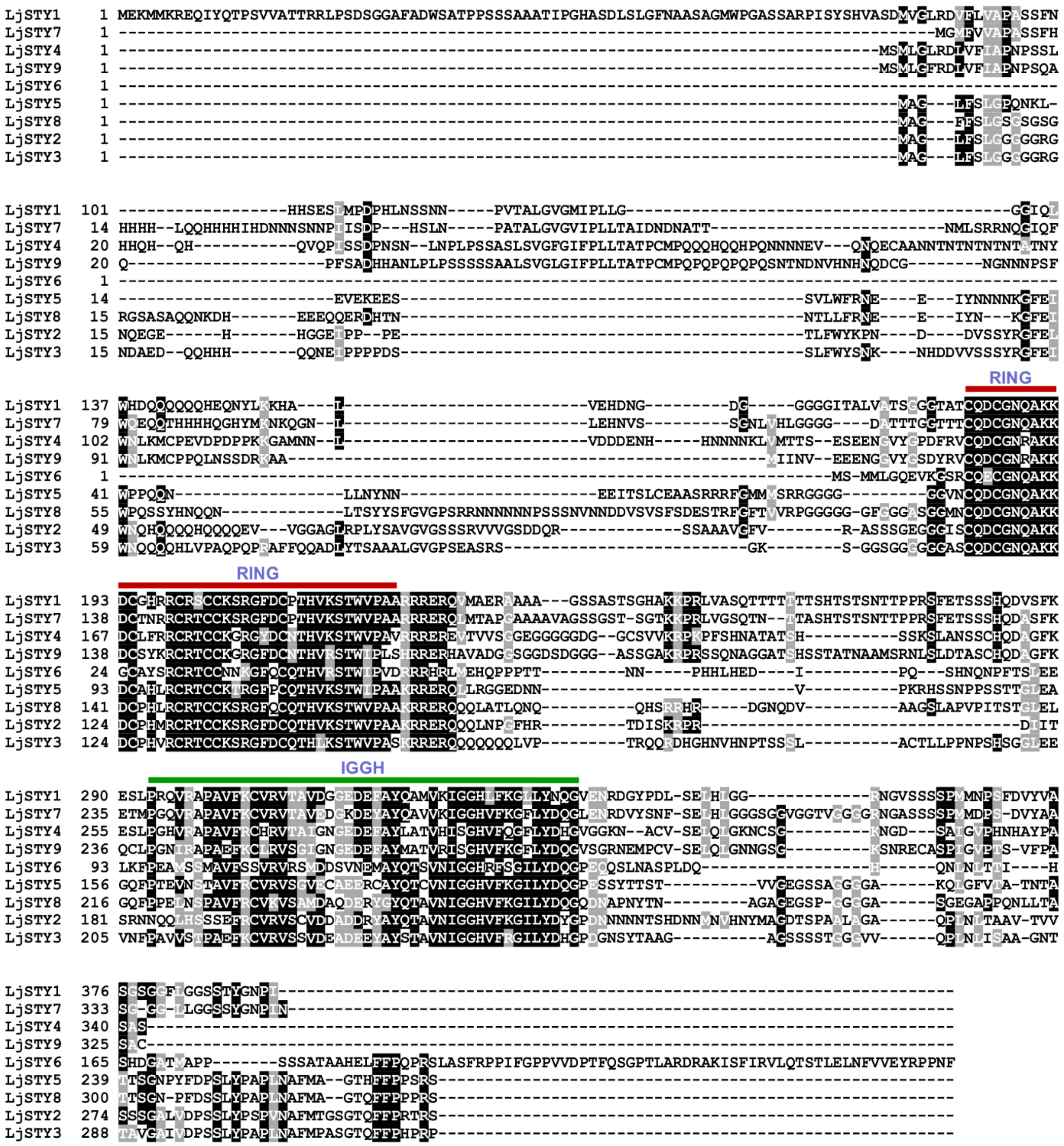

Nine predicted *L. japonicus* STY proteins were aligned with Clustal Omega using the default settings. The BoxShade Server version 3.21 was used to generate the final output. A threshold of ≥ 50% conservation was used. Black shading indicates identical residues, whereas gray indicates presence of conservative substitutions. The following accession numbers refer to the *L. japonicus* protein sequences used: (1) LjSTY1 (Lj6g3v0959410), LjSTY2 (Lj0g3v0059359),

LjSTY3 (Lj2g3v1728900), LjSTY4 (Lj3g3v0766120), LjSTY5 (Lj1g3v2140900), LjSTY6 (Lj3g3v3376040), LjSTY7 (Lj2g3v3044220), LjSTY8 (Lj5g3v0155490)¸ and LjSTY9 (Lj0g3v0258549). The predicted SHI/STY proteins share two evolutionary conserved domains, RING zinc-finger and IGGH, which are highlighted by red and green lines, respectively.

Fig. S2. The putative RING zinc finger domain.

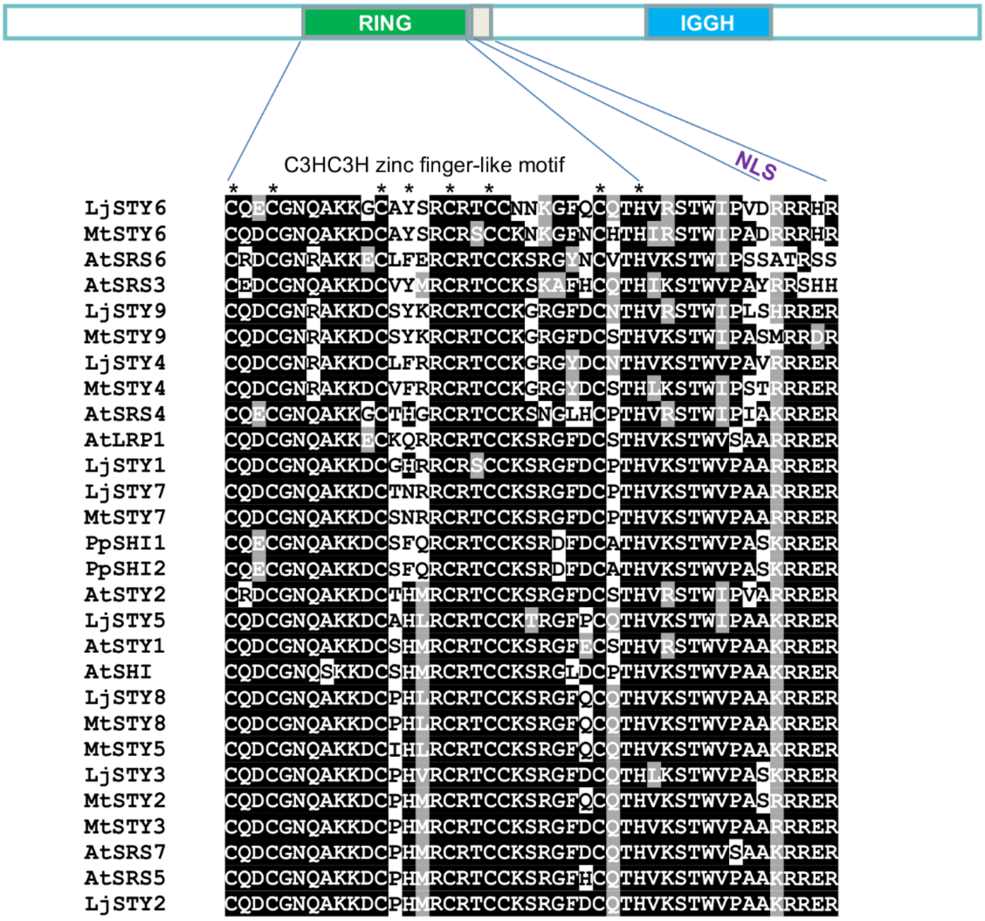

The putative RING zinc finger domain is highly conserved between *Lotus japonicus*, *Medicago truncatula*, *Arabidopsis thaliana* and *Physcomitrella patens* SHI/STY proteins. The domain sequence alignment was generated using the Clustal Omega program with default settings. The BoxShade Server version 3.21 was used to generate the final output. A threshold of ≥ 50% conservation was used. Black shading indicates identical residues, whereas gray indicates presence of conservative substitutions. The accession numbers for SHI/STY proteins are provided in the legend to Fig. 1. Accession numbers for SHI/STY proteins of *P. patens* are PpSHI1 (XP_024359764) and PpSHI2 (XP_024402501).

Fig. S3. Predicted IGGH domain.

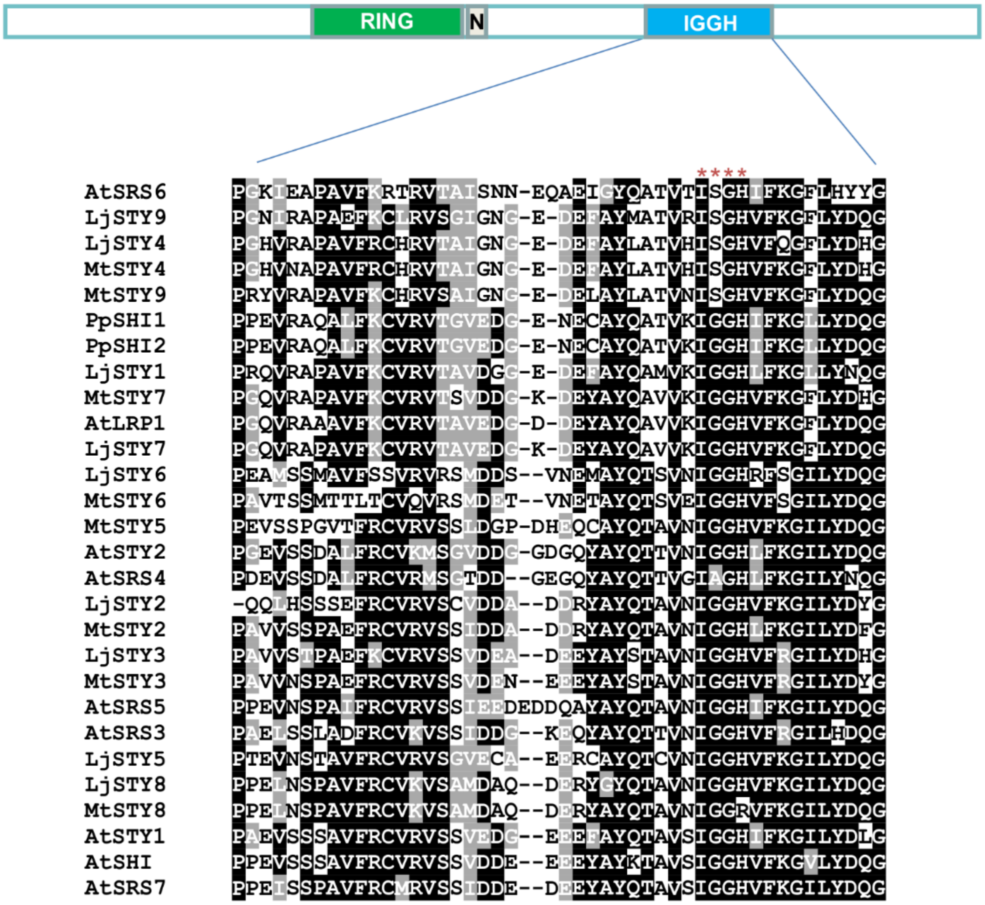

The IGGH domain is highly conserved between *Lotus japonicus*, *Medicago truncatula*, *Arabidopsis* and *Physcomitrella patens* SHI/STY proteins. The domain sequence alignment was generated using the Clustal Omega program with default settings. The BoxShade Server version 3.21 was used to generate the final output. A threshold of ≥ 50% conservation was used. Black shading indicates identical residues, whereas gray indicates presence of conservative substitutions.

Fig. S4. *Lotus japonicus* mutant *sty* alleles.

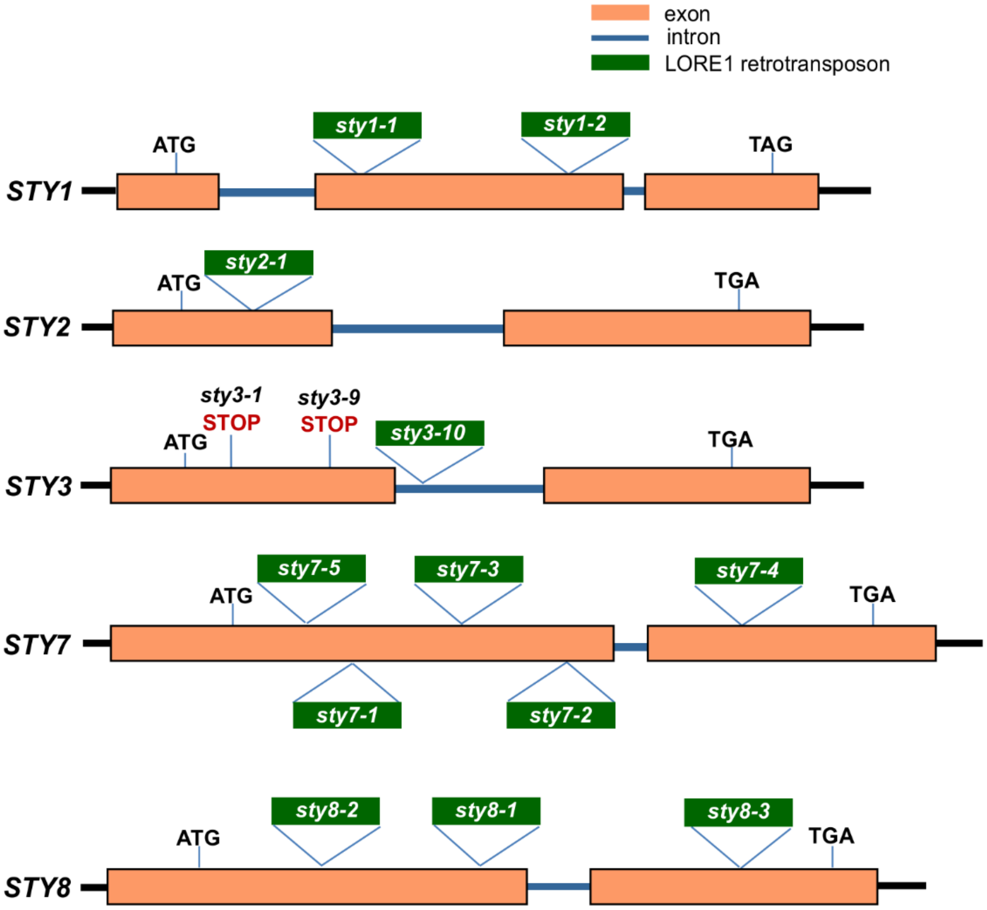

Schematic structures of five *Lotus japonicus STY* genes are shown with the approximate positions of LORE1 retrotransposon insertions (green boxes) or point mutations indicated. Names in the green boxes denote corresponding mutant alleles. Note that, for example, *sty1-1* and *sty1-2* represent two different *sty1* mutant alleles, each present in independent *L. japonicus* mutant lines. The same concept applies to all other *STY* loci. For *STY3*, two point mutations, *sty3-1* and *sty3-9*, which are predicted to generate premature stop codons (STOP), were identified using a TILLING approach (Perry et al., 2009). ATG and TGA correspond to predicted locations of translation initiation and termination signals, respectively.

Fig. S5. *Lotus japonicus* single *sty* mutants have only very subtle symbiotic defects.

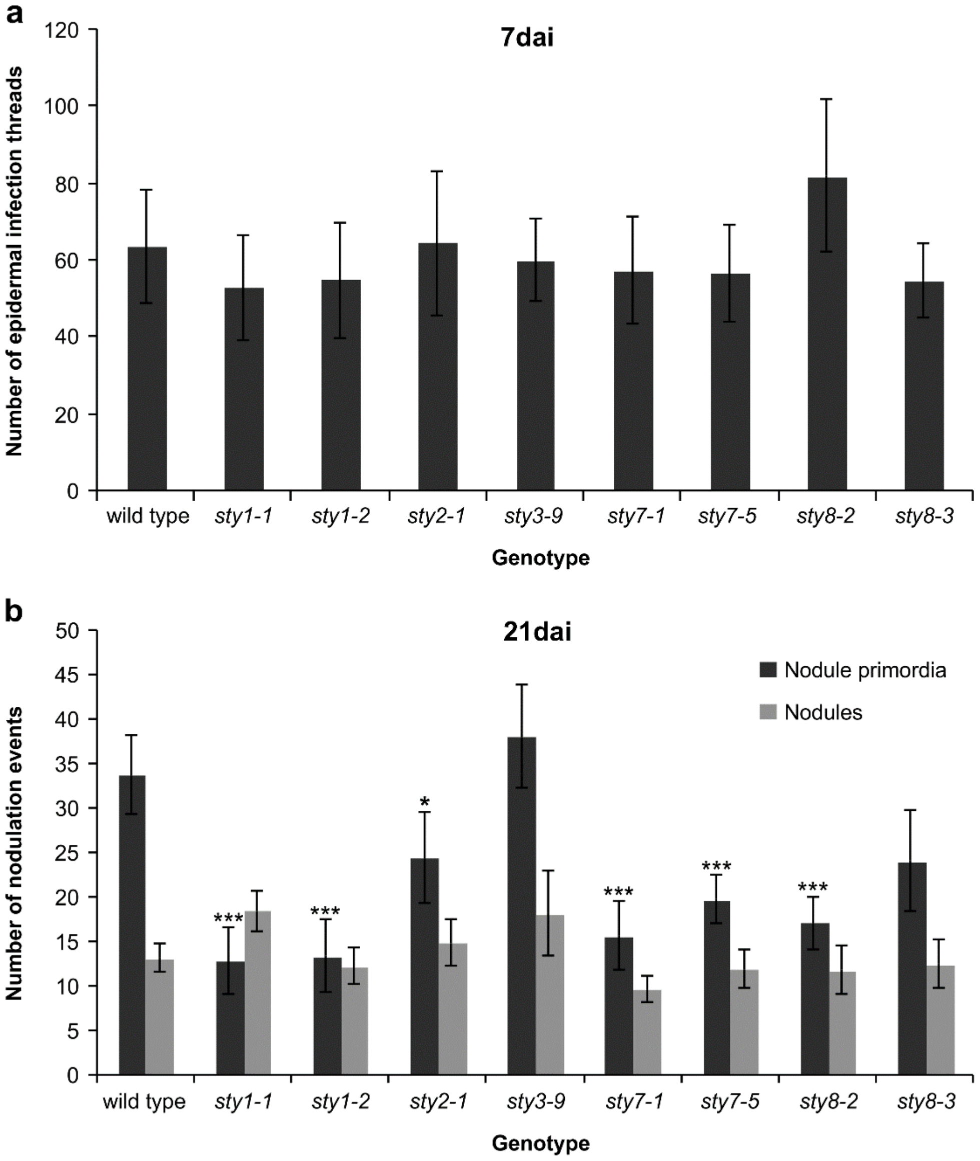

(**a**) Epidermal infection threads were scored in *L. japonicus* wild type and the selected *sty* mutants at 7 dai with *M. loti* strain NZP2235 carrying the *hemA:LacZ* reporter cassette (**b**) The same *sty* mutant lines were evaluated with regard to number of nodule primordia and nodules, which were scored 21 dai with *M. loti*. Note that where available, two independent mutant lines carrying different *sty* alleles (e.g. *sty1-1* and *sty1-2*) were used. Ten individuals were scored for each genotype and averages ± 95% confidence interval are given. Asterisks (*) denote significant differences from wild type (Dunnett's test; *P < 0.05; ***P < 0.001).

Fig. S6. Mutations at most *STY* loci affect non-symbiotic plant growth.

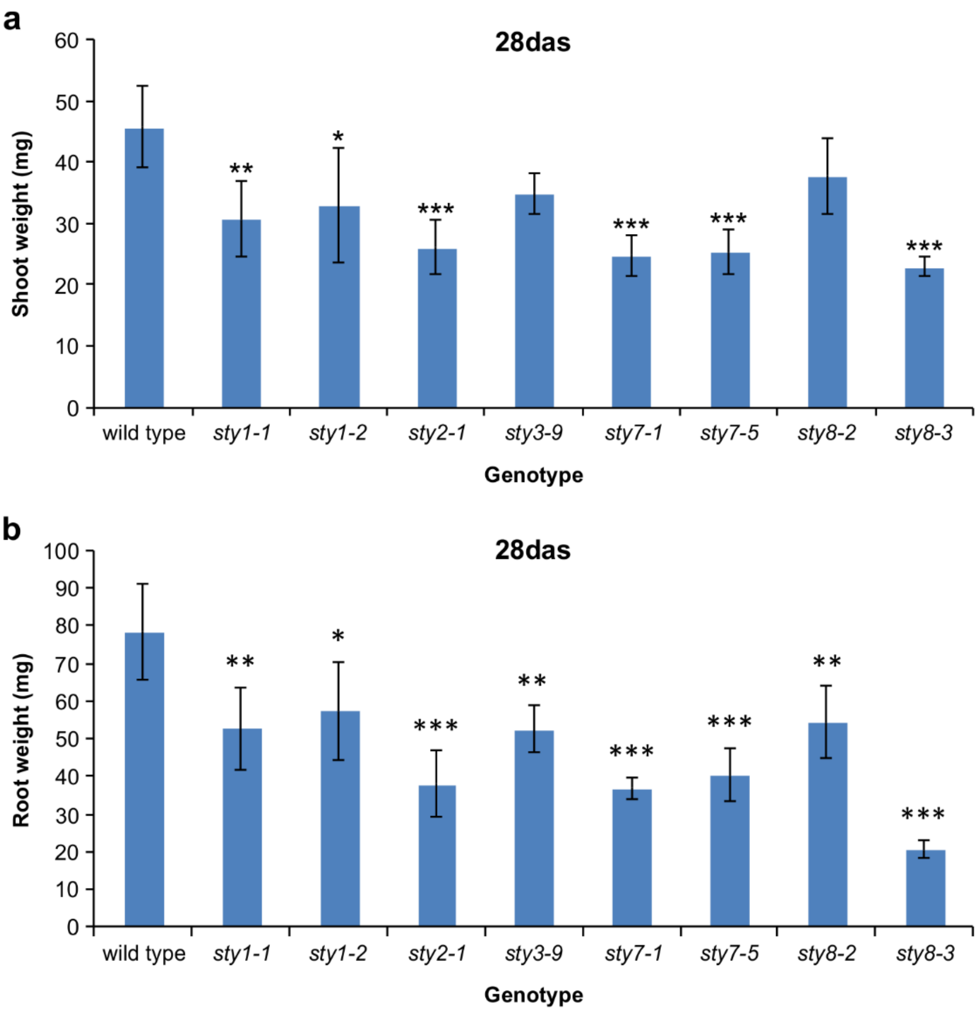

Plants were grown under sterile conditions, in the absence of *M. loti*, and the fresh shoot (**a**) and root weight (**b**) were measured 28 days after sowing (das). Ten plants were scored for each genotype. Averages ± 95% confidence intervals are given. Asterisk denotes a significant difference from the wild-type control (Dunnett's test; *P <0.05; **P <0.01; *** P < 0.01).

Fig. S7. Primary sequence conservation between predicted *Lotus japonicus* YUCCA proteins.

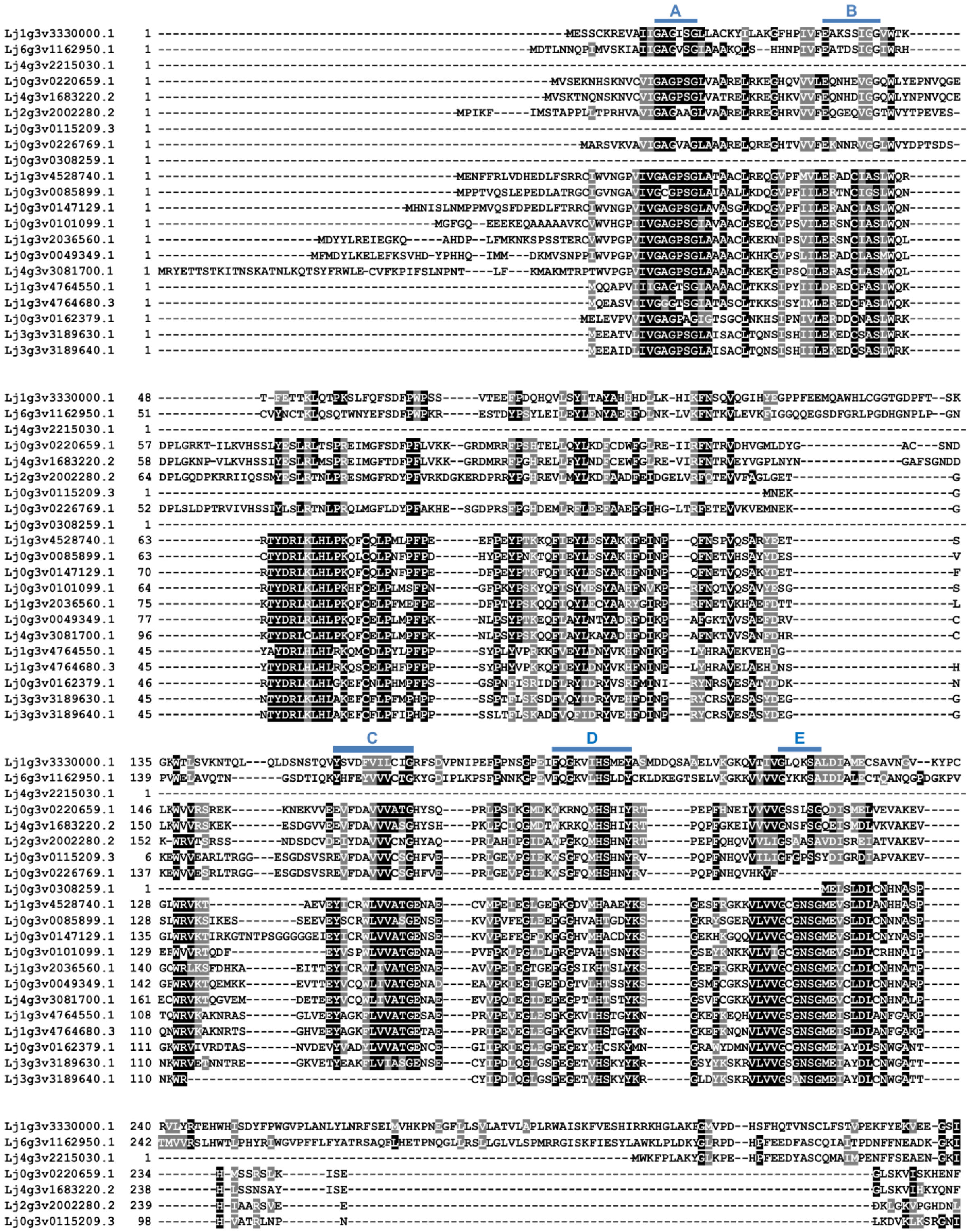

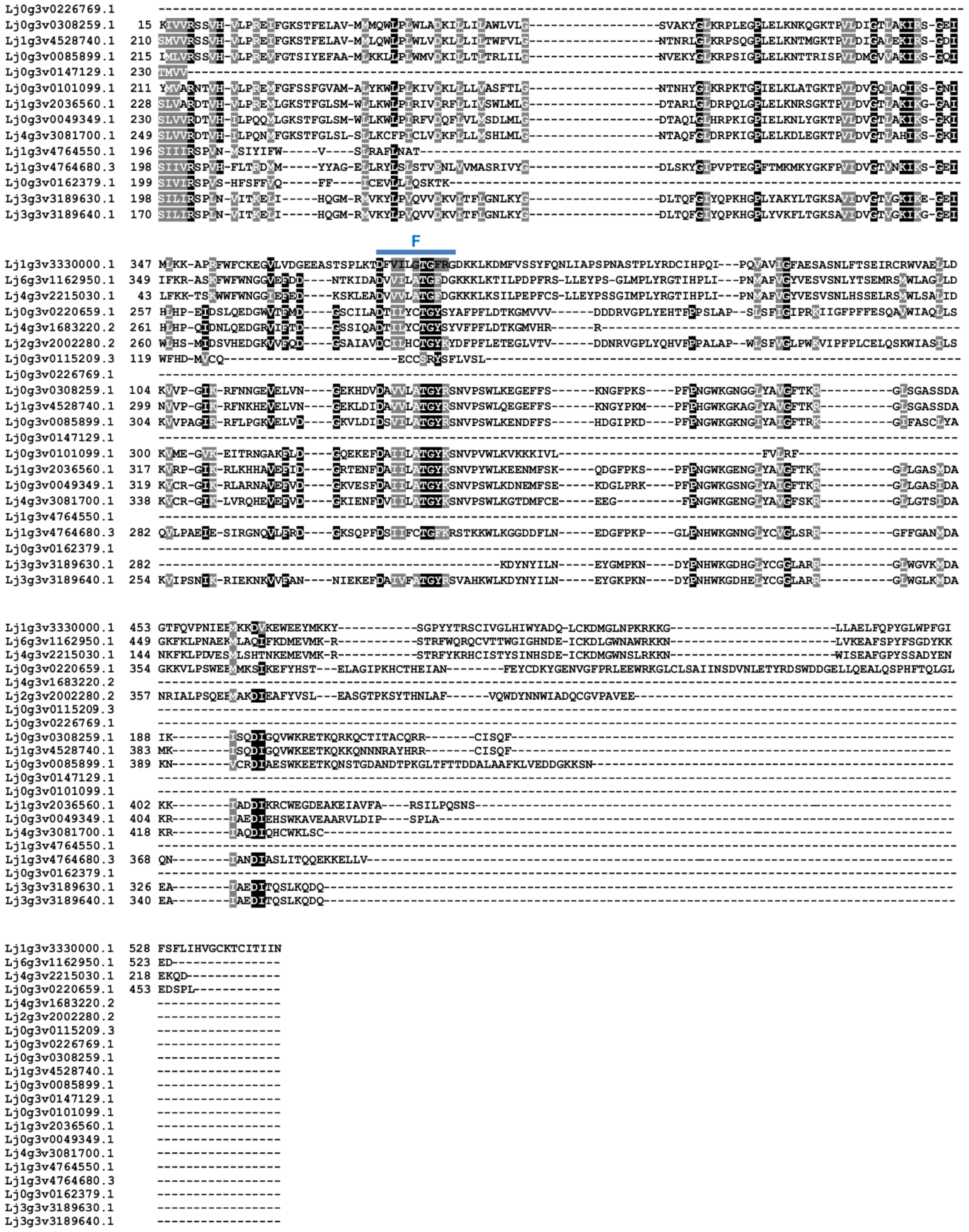
F

Twenty one predicted *L. japonicus* YUCCA-like proteins were aligned with Clustal Omega using the default settings. The BoxShade Server version 3.21 was used to generate the final output. A threshold of ≥ 50% conservation was used. Black shading specifies identical residues, whereas gray indicates presence of conservative substitutions. Letters A to F denote relative conserved regions present in YUCCA-like flavin monooxygenases (Yan et al., 2016).

Fig. S8. Relationship tree between predicted *Lotus japonicus* and *Medicago truncatula* YUCCA-like proteins.

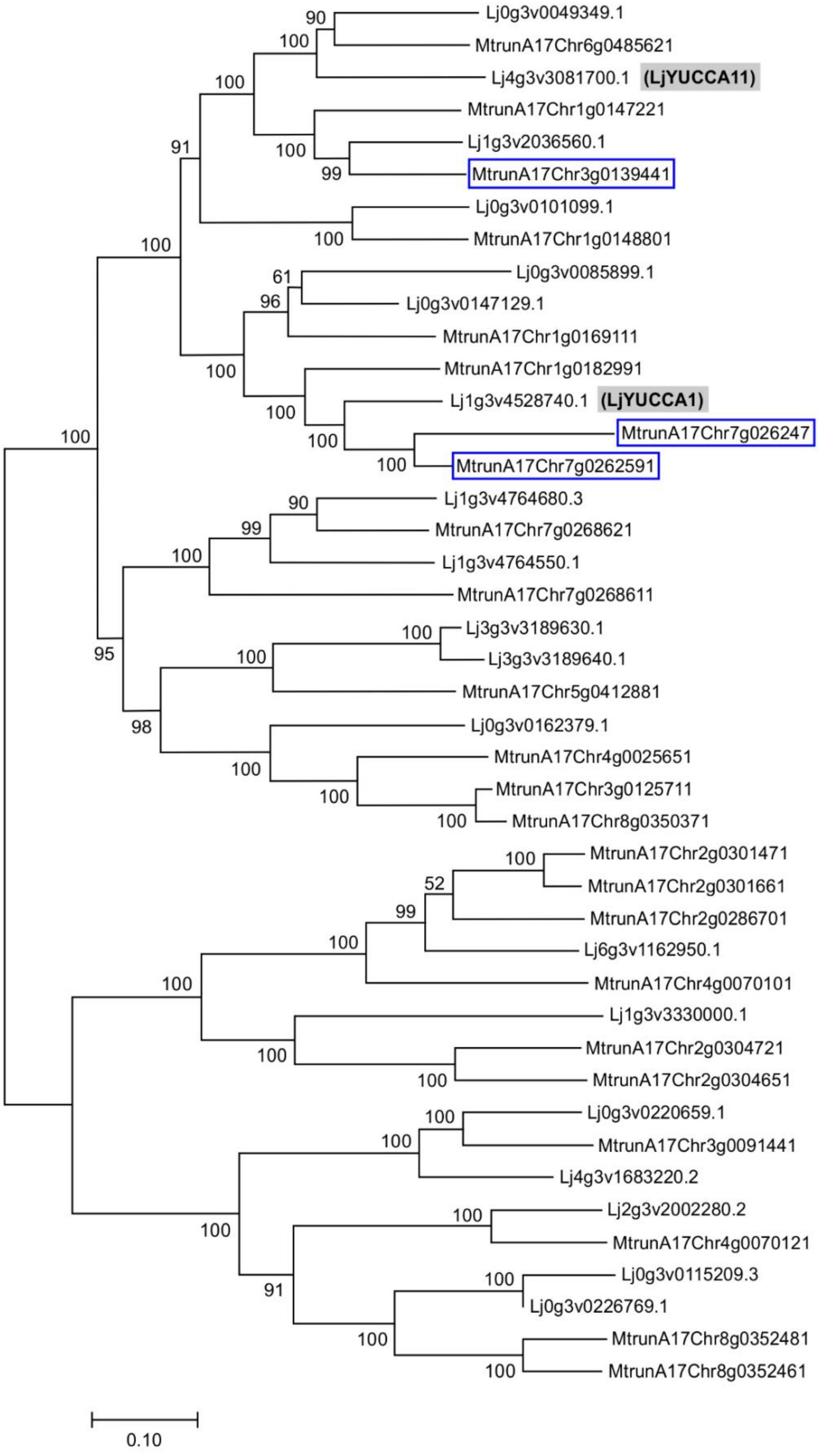

The protein sequences were aligned with ClustalW and the tree was generated using MEGA 7 (Molecular Evolutionary Genetics Analysis) software and neighbor-joining method with bootstrap replicates of 1000. Note that *L. japonicus* Lj0g3v0308259.1 and Lj4g3v2215030.1 and *M. truncatula* MtrunA17Chr3g0129991 and MtrunA17Chr7g0250581 were not included in the tree due to only partially available sequence information. Blue outlines highlight three *M. truncatula* proteins, where the corresponding genes were previously shown to be upregulated in response to rhizobial inoculation or NF application (Larrainzar et al., 2015; Schiessl et al., 2019).

Figure S9. *Lotus japonicus NF-YAs* function partially redundantly to regulate nodule organogenesis.

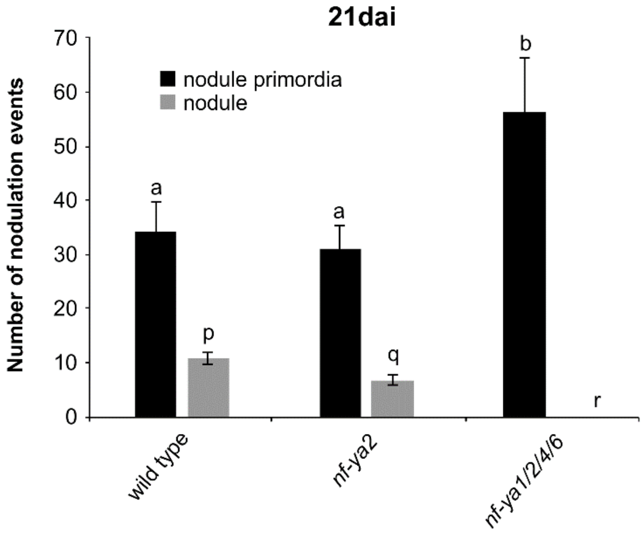

Scores of nodulation events (nodule primordia and nodules) at 21 dai are given. The means ± 95% confidence intervals are presented for 15 individuals per genotype. Asterisks denote significant differences for a pair-wise comparison (The Student's t-test; *** P < 0.001).

Table S1. Primers used in this study.

Name of primer	Primer sequences (5'-3')
**Genotyping primers for *sty3* TILLING alleles**	
sty3-1-F	TCTTCTTACAGAGGCTTTGAGATATGGAACCACCGG
sty3-1-R	AGAGTGCAAGCGAGTGATGAA
sty3-9-F	CGCTAGGTGTTGGGCCGTCG
sty3-9-R	ATCTCTCTGCTGCCTTGTAGGAACCAGCT
**Sequencing primers for *sty3-1* and *sty3-9***	
sty3-1-seq-F	ATGGCGGGTTTATTCTCACTAG
sty3-1-seq-R	TCCTCCTCCACCTCCACTTC
sty3-1-seq-R1	AGCAAGTTCTGCACCTCACA
sty3-9-seq-F	GAAGTGGAGGTGGAGGAGGA
sty3-9-seq-R	TGCACTCTGCAGGATTCTGTT
sty3-9-seq-F1	TGTGAGGTGCAGAACTTGCT
sty3-9-seq-R1	ACACGCAACCGATCTTCCTT
**qRT-PCR Primers for *STYs***	
STY1-qPCR-F	ACGCTCTTGTTGAGCACGAT
STY1-qPCR-R	CTCCTGTGTCCGCAATCCTT
STY2-qPCR-F	GGTACAAACCAAACGACGATG
STY2-qPCR-R	CTTAACCCTGCTCCTCCAACT
STY3-qPCR-F	GGCCAGACGGCAATAGTTACAC
STY3-qPCR-R	GAGCCGGATACAAGGAAGAAGG
STY4-qPCR-F	TGGAACCTCAAAATGTGCCC
STY4-qPCR-R	TCAGGACCGTAAACACCGTT
STY5-qPCR-F	TGATGATGAGTAGGCGTGGTG
STY5-qPCR-R	TGTGGTGGAGGATGGAGGATT
STY6-qPCR-F	ATGGCGGTATTCAGCAGTGTT
STY6-qPCR-R	ATCATGGCTGTGGATTGTGG
STY7-qPCR-F	GAGGATGGGAAGGATGAGTATG
STY7-qPCR-R	CTCCACCACCAACAGTACCAC
STY8-qPCR-F	AGGCCAAGACAATGCTCCTA
STY8-qPCR-R	GCGTACCAGCCATGAAAGCA
STY9-qPCR-F	GTGGCTGATGGTGGTAGTGG
STY9-qPCR-R	TTAGTGGCGGTGGAACTGTG
UBQ-F	ATGTGCATTTTAAGACAGGG
UBQ-R	GAACGTAGAAGATTGCCTGAA
PP2A-F	GTAAATGCGTCTAAAGATAGGGTCC
PP2A-R	ACTAGACTGTAGTGCTTGAGAGGC
ATPs-F	AACACCACTCTCGATCATTTCTCTG
ATPs-R	CAATGTCGCCAAGGCCCATGGTG

**Genotyping primers for *STY3::SRDX* transgene**	
STY3-SRDX-F	AAGTGAGAGAAGAATGGCGGG
STY3-SRDX-R	GCCAAGGATGGATTTCCTAAGC
**qRT-PCR Primers for *YUCCAs***	
YUCCA1-qPCR-F	GAGTTGGCGGTTATGATGCTG
YUCCA1-qPCR-R	CAGGGGTTTTTCCCATTGTGTT
YUCCA11-qPCR-F	ACAGCACGAAGTGGAGTTTG
YUCCA11-qPCR-R	AAGCAGGCCACGTTTAGAGA
***YUCCA1* and *YUCCA11* promoter amplification primers**	
pYUCCA1-DT-F	CACCTCATCCACTGTCTGTTAAG
pYUCCA1-DT-R	TTTGAATTTTGTGTGTGTTATG
pYUCCA11-DT-F	CACCAATGCAAGACATTGAC
pYUCCA11-DT-R	ATGAATTGAAAACCAAACATATAC
***YUCCA1* and *YUCCA11* promoter sequencing primers**	
pYUCCA1-F1	ATAACCTCCGATCCACTTC
pYUCCA1-F2	TGGAGTGTTATTCTAACA
pYUCCA1-F3	TACGTGTCAGTATGTCCTGC
pYUCCA1-F4	ATTTCTTCCATACCACTTG
pYUCCA1-F5	TCTCACAGAATATAGTGT
pYUCCA1-F6	TCAATCCAACACTCTCAAC
pYUCCA1-R1	CAACGATGCAGTGGAGCA
pYUCCA1-R2	ACATCACAGTCCTCCTCATTC
pYUCCA1-R3	ATCTATACAGTCTCTCTT
pYUCCA1-R4	GTATATGCATTTTCCATGCAC
pYUCCA1-R5	GACAATCCTTTGGTTATGTATG
pYUCCA11-F1	GTACTAGTGTCGCTACCAGATTG
pYUCCA11-F2	CGTCACATAGTTCTTGTCAGGAG
pYUCCA11-F3	TCAAAGCAAGGAATTTGTGAC
pYUCCA11-F4	CTATGAGTCATTCAAGCAATA
pYUCCA11-F5	CAAGTAGTCAGTTGTAGTGTG
pYUCCA11-F6	CAGTCCTTTCTTGAGGACAGTC
pYUCCA11-R1	GCATTGCTCGTATTAGGAG
pYUCCA11-R2	GTGACAGCCTTATATTTCGTC
pYUCCA11-R3	GTGACAGAATCTTAGAGAG
pYUCCA11-R4	CGAATCATGGATCAGGTACCT
pYUCCA11-R5	CACACAGACATTGGGAGTCAG
pYUCCA11-R6	GAGCACACAGCAGGAAGCAATGT
pYUCCA11-R7	CTCGTCACTCAGTGCATGT

Table S2: Expression of four *Lotus japonicus STY* genes is significantly upregulated during early stages of symbiosis.

Gene name	Gene ID	log2FC	P-value	FDR-value
STY1	Lj6g3v0959410	2.46	4.774E-15	3.72E-12
STY2	Lj0g3v0059359	2.69	7.994E-15	5.95E-12
STY3	Lj2g3v1728900	2.43	2.22E-16	2.02E-13
STY4	Lj3g3v0766120	-0.29	0.442	0.999
STY5	Lj1g3v2140900	0.99	0.122	0.999
STY6	Lj3g3v3376040	-0.57	0.598	0.999
STY7	Lj2g3v3044220	1.05	3.5E-07	6.58E-05
STY8	Lj5g3v0155490	1.43	0.001	0.068
STY9	Lj0g3v0258549	0.56	0.254	0.999

The wild-type, un-inoculated roots and roots of the same age collected 4 dai with *M. loti* were analyzed using next-generation RNA sequencing (BioProject ID PRJNA630938**;** http://www.ncbi.nlm.nih.gov/bioproject/630938**)**. Of the nine *L. japonicus STY* mRNAs, *STY1*, *STY2*, *STY3*, and *STY7* were found to be significantly (FDR<0.05) upregulated 4 dai with *M. loti* (highlighted in gray). Log2FC: log2 fold change from the corresponding, un-inoculated wild-type roots; P-value: uncorrected p-value; FDR-value: false discovery rate.

Tables S3. Analysis of *Medicago truncatula STY* gene expression.

a

		RNAseq after laser caption of nodule zones						
**Gene name**	**MtV5 id**	**%FI**	**%FIID**	**%FIIP**	**%IZ**	**%ZIII**	**Upregulated 48h post inoculation (Larrainzar et al. 2015)**	**FC Nodule/Root (Roux et al. 2014)**
MtSTY2	MtrunA17Chr8g0372461	52.7	19.1	5.5	11.3	11.4	Yes	85.6
MtSTY3	MtrunA17Chr5g0404781	70.9	16.7	5.3	5.0	2.2	Yes	8
MtSTY4	MtrunA17Chr3g0082511	19.5	16.5	3.3	45.9	14.9	Yes	3.84
MtSTY5	MtrunA17Chr3g0142171	100.0	0.0	0.0	0.0	0.0	No	nd in roots
MtSTY6	MtrunA17Chr4g0035591	57.9	23.3	18.8	0.0	0.0	No	0.82
MtSTY7	MtrunA17Chr5g0441921	68.6	16.8	3.3	4.9	6.5	Yes	4.22
MtSTY8	MtrunA17Chr1g0155791	94.9	5.2	0.0	0.0	0.0	Yes	5.08
MtSTY9	MtrunA17Chr8g0353111	5.6	6.3	11.7	37.1	39.4	No	302.03

b

		Expression in *nf-ya1-1* mutant vs wild type			
**Gene name**	**MtV5 id**	**4dpi (log2FC)**	**4dpi (p value)**	**10dpi (logFC)**	**10dpi (p value)**
MtSTY2	MtrunA17Chr8g0372461	1.61	4.02E-05	0.11	5.65E-01
MtSTY3	MtrunA17Chr5g0404781	0.66	3.70E-03	0.72	2.76E-04
MtSTY4	MtrunA17Chr3g0082511	0.90	8.20E-05	0.43	5.34E-02
MtSTY5	MtrunA17Chr3g0142171	nd	nd	nd	nd
MtSTY6	MtrunA17Chr4g0035591	nd	nd	0.29	3.52E-01
MtSTY7	MtrunA17Chr5g0441921	0.81	1.72E-06	0.77	1.53E-04
MtSTY8	MtrunA17Chr1g0155791	0.99	1.96E-03	0.82	7.52E-04
MtSTY9	MtrunA17Chr8g0353111	2.25	3.74E-09	1.94	3.78E-11

(**a**) Compilation of RNAseq expression data for *M. truncatula STY* genes, as based on Roux et al. (2014) and Larrainzar et al. (2015), is shown. Percentages (%) of RNA reads from five different nodule regions are given. FI, nodule meristematic zone; FIID, nodule meristem distal zone; FIIP, nodule meristem proximal zone; IZ, interzone; ZIII, nitrogen-fixation zone, as in Roux et al. (2014). (**b**) RNAseq expression analysis, comparing *M. truncatula* wild-type and *Mtnf-ya1-1* transcriptomes 4 and 10 days post inoculation with *Sinorhizobium meliloti*. The Benjamini-Hochberg (BH) procedure was used to calculate the P-value of the false discovery rate. Gray boxes represent the genes for which this P-value is <5%. nd= not detected.

Table S4. List of *sty* alleles carrying a LORE1 insertion, as identified from the Lotus Base information portal (https://lotus.au.dk/).

Mutant allele	Gene ID	LORE1 Line number
sty1-1	Lj6g3v0959410	30052423
sty1-2	Lj6g3v0959410	30032212
sty2-1	Lj0g3v0059359	P1687
sty3-10	Lj2g3v1728900	30010699
sty4-1	Lj3g3v0766120	30083039
sty4-2	Lj3g3v0766120	30007756
sty5-1	Lj1g3v2140900	30115636
sty5-2	Lj1g3v2140900	30120414
sty5-3	Lj1g3v2140900	30089004
sty6-1	Lj3g3v3376040	30136178
sty7-1	Lj2g3v3044220	30060832
sty7-2	Lj2g3v3044220	30074305
sty7-3	Lj2g3v3044220	30084924
sty7-4	Lj2g3v3044220	30092737
sty7-5	Lj2g3v3044220	30097763
sty8-1	Lj5g3v0155490	30088537
sty8-2	Lj5g3v0155490	30089290
sty8-3	Lj5g3v0155490	30109651
sty9-1	Lj0g3v0258549	30034946
sty9-2	Lj0g3v0258549	30109475
sty9-3	Lj0g3v0258549	30058721

Table S5. Segregation of the *proNF-YA1:STY3::SRDX* transgene in T1 populations, *STY3::SRDX5* and *STY3::SRDX6*, derived from two independent T0 plants.

a

Transgenic T0 Plants	Total T1 plants	*pNF-YA1:STY3::SRDX* -		*pNF-YA1:STY3::SRDX* +	
		Nod +	Nod -	Nod +	Nod -
STY3::SRDX5	85	23	0	0	62
STY3::SRDX6	72	21	0	0	51

b

STY::SRDX5	*pNF-YA1:STY3::SRDX* -	*pNF-YA1:STY3::SRDX* +
Observed no. (O)	23	62
Expected no. (E)	21.25	63.75
$\chi 2$ calculated	0.192156863	
$\chi 2$ critical (P = 0.05)	3.841	

STY3::SRDX6	*pNF-YA1:STY3::SRDX* -	*pNF-YA1:STY3::SRDX* +
Observed no. (O)	21	51
Expected no. E	18	54
$\chi 2$ calculated	0.666666667	
$\chi 2$ critical (P = 0.05)	3.841	

(**a**) Number of plants segregating the *pNF-YA1:STY3::SRDX* transgene and the corresponding nodulation phenotypes. (+/-) symbols denote presence and absence of the transgene and nodules (Nod), respectively. (**b**) The corresponding Chi-square ($\chi 2$) test results for segregation of the transgene in the two T1 populations (P = 0.05).

Table S6. *YUCCA11* is regulated upon *Mesorhizobium loti* inoculation**.**

Gene ID	log2F	P-value	FDR-value
Lj1g3v4528740.1 (LjYUCCA1)	0.67	0.0036	0.16
Lj0g3v0049349.1	0.26	0.4651	0.1
Lj0g3v0085899.1	-0.44	0.0295	0.64
Lj0g3v0308259.1	0.07	0.8036	0.1
Lj3g3v3189630.1	nd	nd	nd
Lj3g3v3189640.1	nd	nd	nd
Lj1g3v2036560.1	-0.76	0.0070	0.25
Lj1g3v4764550.1	-0.09	0.7478	0.1
Lj1g3v4764680.3	0.01	0.9679	0.1
Lj0g3v0101099.1	3.18	0.0946	0.1
Lj4g3v3081700.1 (LjYUCCA11)	8.31	0.0006	0.04
Lj0g3v0115209.3	0.23	0.2437	0.1
Lj0g3v0147129.1	0.57	0.6486	0.1
Lj0g3v0162379.1	nd	nd	nd
Lj0g3v0220659.1	-0.01	0.9649	0.1
Lj0g3v0226769.1	0.13	0.6178	0.1
Lj1g3v3330000.1	-1.15	0.0712	0.1
Lj2g3v2002280.2	-0.42	0.1837	0.1
Lj4g3v1683220.2	-0.20	0.3290	0.1
Lj4g3v2215030.1	0.03	0.9320	0.1
Lj6g3v1162950.1	-0.15	0.2437	0.1

Wild-type, un-inoculated roots and those of the same age collected 4 dai with *M. loti*, were analyzed using next-generation RNA sequencing (BioProject ID PRJNA630938; http://www.ncbi.nlm.nih.gov/bioproject/630938). Of the 21 *YUCCA*-like genes, only *YUCCA11* was found to be significantly (FDR<0.05) regulated by *M. loti* inoculation (highlighted in yellow) Log2FC: log2 fold change; P-value: uncorrected P-value; FDR-value: false discovery rate (corrected P value), nd= not detected.

Table S7. A list of mutant *nf-ya* alleles used in this study. Note that information about the corresponding LORE1 insertion lines can be found at Lotus Base (https://lotus.au.dk/).

Mutant allele	Gene ID	LORE1 line number	Remarks
nf-ya1-2	Lj5g3v0841080	N/A	EMS mutant (Hossain et al., 2016)
nf-ya2	Lj6g3v0647470	30162806	Insertion in the first exon
nf-ya4	Lj1g3v4752710	30092825	Insertion in the first exon
nf-ya6	Lj3g3v0338970	30065649	Insertion in the third exon

Table S8. Levels of *NF-YA* mRNAs in 11 day-old un-inoculated *Lotus japonicus* roots.

Gene name	Gene ID	Average TPM
LjNF-YA1	Lj5g3v0841080	0.5
LjNF-YA2	Lj6g3v0647470	27.8
LjNF-YA3	Lj4g3v2179250	3.3
LjNF-YA4	Lj1g3v4752710	89.7
LjNF-YA5	Lj3g3v2657800	12.1
LjNF-YA6	Lj3g3v0338970	18.2
LjNF-YA7	Lj2g3v3336090	11.6
LjNF-YA8	Lj0g3v0252369	6.4

TPM: transcripts per million, as determined using 11day-old un-inoculated roots (BioProject ID PRJNA630938; http://www.ncbi.nlm.nih.gov/bioproject/630938).

References

Perry J, Brachmann A, Welham T, Binder A, Charpentier M, Groth M, Haage K, Markmann K, Wang TL, Parniske M. 2009. TILLING in *Lotus japonicus* identified large allelic series for symbiosis genes and revealed a bias in functionally defective ethyl methanesulfonate alleles toward glycine replacements. *Plant Physiology* **151**: 1281-1291.

Yan S, Che G, Ding L, Chen Z, Liu X, Wang H, Zhao W, Ning K, Zhao J, Tesfamichael K, et al. 2016. Different cucumber *CsYUC* genes regulate response to abiotic stresses and flower development. *Scientific Reports* **6:** 20760.

Roux B, Rodde N, Jardinaud MF, Timmers T, Sauviac L, Cottret L, Carrere S, Sallet E, Courcelle E, Moreau S, et al. 2014. An integrated analysis of plant and bacterial gene expression in symbiotic root nodules using laser-capture microdissection coupled to RNA sequencing. *Plant Journal* **77**: 817-837.

Larrainzar E, Riely BK, Kim SC, Carrasquilla-Garcia N, Yu HJ, Hwang HJ, Oh M, Kim GB, Surendrarao AK, Chasman D, et al. 2015. Deep sequencing of the *Medicago truncatula* root transcriptome reveals a massive and early interaction between nodulation factor and ethylene signals. *Plant Physiology* **169**: 233-265.

Schiessl K, Lilley JLS, Lee T, Tamvakis I, Kohlen W, Bailey PC, Thomas A, Luptak J, Ramakrishnan K, Carpenter MD, et al. 2019. NODULE INCEPTION recruits the lateral root developmental program for symbiotic nodule organogenesis in *Medicago truncatula*. *Current Biology* **29**: 3657-3668 e3655.
